# Quality of Life and Patient-Reported Outcomes During First-Line Chemotherapy in Older Patients with De Novo Metastatic Gastrointestinal Cancers: A Prospective Longitudinal Study

**DOI:** 10.3390/healthcare14203458

**Published:** 2026-10-15

**Authors:** Hatice Ayyıldız Sevim, Hayriye Şahinli, Galip Can Uyar, Selahattin Çelik, Kadriye Bir Yücel, Osman Sütcüoğlu

**Affiliations:** 1 Department of Medical Oncology, Ankara Etlik City Hospital, Ankara 06170, Türkiye; g.can_uyar@hotmail.com (G.C.U.); drcelikselahattin@gmail.com (S.Ç.); kadriyebiryucel@gmail.com (K.B.Y.); 2 Department of Medical Oncology, Ankara Etlik City Hospital, University of Health Sciences, Ankara 06170, Türkiye; dr.hayriye@hotmail.com; 3 Department of Medical Oncology, Gazi University, Ankara 06500, Türkiye; sutcuogluo@gmail.com

**Keywords:** older adults, gastrointestinal cancer, quality of life, geriatric assessment, patient-reported outcomes

## Abstract

Background/Objectives: This study was conducted to prospectively evaluate quality of life (QoL), patient-reported outcomes, and geriatric domains during the first 3 months of first-line systemic therapy in older patients with de novo metastatic gastrointestinal cancers. Methods: This prospective longitudinal study included 90 patients aged ≥65 years receiving first-line chemotherapy-based systemic therapy. Assessments were performed at baseline, Month 1, and Month 3 using validated QoL, sleep, anxiety, cognitive, and nutritional instruments. The primary endpoint was change in EORTC QLQ-C30 Global Health Status/QoL from baseline to Month 3. Treatment tolerability, initial radiologic response, and exploratory survival outcomes were also assessed. Results: Mean Global Health Status/QoL increased from 68.1 at baseline to 71.0 at Month 3; the model-estimated mean change was +3.0 points (95% CI, −0.3 to 6.2; *p* = 0.071). Fatigue and appetite loss improved significantly, and normal nutritional status increased from 31.1% to 62.2% (*p* < 0.001). Clinically meaningful QoL improvement and deterioration occurred in 38.9% and 18.9%, respectively. Treatment intolerance occurred in 46.7%. Disease control was associated with more favorable QoL change than progressive disease (difference, 17.8 points; 95% CI, 6.0–29.7; FDR-adjusted *p* = 0.008). Baseline nutritional impairment (adjusted HR, 3.58) and poor sleep quality (adjusted HR, 3.30) were associated with shorter progression-free survival. Conclusions: Mean global QoL did not change significantly during the first 3 months, while several symptom and nutritional domains improved. More favorable QoL trajectories were observed with disease control, and baseline nutritional impairment and poor sleep quality were associated with shorter progression-free survival.

## 1. Introduction

Older adults with metastatic gastrointestinal cancers constitute a clinically challenging population in whom treatment decisions should consider not only disease control but also treatment tolerance, functional status, and quality of life (QoL) [1,2]. Randomized evidence in vulnerable older patients with metastatic colorectal cancer has demonstrated the relevance of QoL and physical functioning as important outcomes during systemic treatment [1]. Chronological age and routine oncology assessments may not adequately identify age-related vulnerabilities; accordingly, ASCO recommends geriatric assessment covering physical and cognitive function, emotional health, comorbidities, polypharmacy, nutrition, and social support in adults aged >65 years receiving systemic cancer therapy [2]. The importance of patient-centered outcomes is further illustrated by a secondary analysis of the GAP70+ trial, in which 71.7% of older adults with incurable cancer prioritized maintaining QoL, whereas only 8.4% prioritized extending survival [3].

Beyond tumor characteristics and treatment regimen, a patient-centered approach to treating older adults with cancer also requires attention to factors that may influence their experience during systemic therapy. Among older adults with newly diagnosed colorectal cancer, moderate-to-severe anxiety was associated with greater frailty and impaired physical and mental health-related quality of life [4]. In a prospective study of patients aged ≥70 years receiving first-line chemotherapy, baseline cognitive impairment and impaired nutritional status were common, and both were independently associated with cognitive impairment during chemotherapy [5]. In patients with gastrointestinal cancers receiving chemotherapy, those with higher sleep-disturbance profiles reported greater anxiety, depressive symptoms, morning and evening fatigue, and pain, together with lower quality-of-life scores [6]. Importantly, the randomized Geriatric Assessment–Driven Intervention (GAIN) trial showed that a multidisciplinary geriatric assessment–based intervention significantly reduced the incidence of grade ≥ 3 chemotherapy-related toxicity in older adults with cancer [7].

In older patients, it is clinically important to characterize not only baseline geriatric status but also how these domains change during treatment. In the EpiReal 75 study, serial geriatric assessments in older patients with colorectal cancer identified changes in functional status during chemotherapy and demonstrated that these changes were associated with quality-of-life outcomes [8]. Similarly, the INTEGERATE trial showed that integrating geriatric assessment into oncology care improved health-related quality of life in older adults initiating systemic anticancer therapy [9]. Nevertheless, prospective longitudinal data jointly evaluating quality of life, patient-reported outcomes, and geriatric domains during treatment remain limited in older adults with metastatic gastrointestinal cancers. Therefore, this study aimed to prospectively evaluate changes in quality of life during the first 3 months of first-line systemic therapy in older adults with de novo metastatic gastrointestinal cancers, together with longitudinal changes in sleep, anxiety, nutritional status, cognitive status, performance status, and body weight, and to additionally assess treatment tolerance, tumor response, and survival outcomes.

## 2. Materials and Methods

### 2.1. Study Design and Participants

This single-center, prospective, longitudinal observational study was conducted at the Department of Medical Oncology, Ankara Etlik City Hospital. Patients were prospectively enrolled between 1 June 2025, and 1 June 2026. Patients aged ≥65 years with de novo metastatic gastrointestinal cancer, defined by the presence of distant metastatic disease at initial diagnosis, who initiated first-line chemotherapy-based systemic therapy as part of routine clinical practice were eligible. All patients received first-line chemotherapy, either alone or in combination with targeted therapy or immunotherapy, according to clinical indications. No patient received targeted therapy or immunotherapy without concomitant chemotherapy.

Eligible primary tumor sites included esophageal, gastric, colorectal, pancreatic, and biliary tract cancers. Patients were required to have received no prior systemic anticancer therapy before initiation of first-line treatment and to be able to provide written informed consent.

Exclusion criteria were age < 65 years, nonmetastatic gastrointestinal cancer, previous systemic oncologic treatment before initiation of first-line therapy, cognitive, neurologic, or psychiatric impairment precluding informed consent or completion of the planned assessments, severe uncontrolled comorbidity precluding systemic treatment or study participation, and inability to complete the planned prospective assessments.

### 2.2. Data Collection and Assessment Schedule

Assessments were performed at initiation of first-line systemic therapy (baseline), at the end of Month 1, and at the end of Month 3. At baseline, demographic and geriatric characteristics, including age, sex, education level, comorbidities, number of chronic medications, history of falls during the previous 6 months, and living arrangement, were recorded. Clinical and disease-related variables included ECOG performance status, body weight and body mass index, primary tumor site, number and sites of metastatic disease, history of primary tumor resection, and characteristics of first-line treatment. Quality of life and patient-reported/geriatric measures were assessed using the EORTC QLQ-C30, Pittsburgh Sleep Quality Index (PSQI), Generalized Anxiety Disorder-7 (GAD-7), Standardized Mini-Mental State Examination (SMMSE), and Mini Nutritional Assessment–Short Form (MNA-SF). Validated Turkish versions of all instruments were used [10,11,12,13,14]. All patient-reported and geriatric assessments were administered face-to-face by the same investigator at baseline, Month 1, and Month 3. All assessments were conducted according to the standardized administration procedures of the respective instruments. During repeated assessments, the investigator was unaware of the results of previous assessments. ECOG performance status and body weight were reassessed at the same three time points. All assessments were performed during routine clinical follow-up, and no additional therapeutic intervention was introduced as part of the study. No standardized nutritional, psychosocial, palliative-care, or rehabilitation interventions were implemented during the 3-month follow-up period.

### 2.3. Assessment Instruments and Scoring

Quality of life was assessed using the European Organisation for Research and Treatment of Cancer Quality of Life Questionnaire Core 30 (EORTC QLQ-C30). Scores were transformed to a 0–100 scale according to standard EORTC scoring procedures. Higher scores on the Global Health Status/QoL and functional scales indicated better health status or functioning, whereas higher scores on symptom scales indicated greater symptom burden. The EORTC QLQ-C30 Global Health Status/QoL score served as the principal quality-of-life measure. The functional and symptom scales were evaluated separately as domain-specific outcomes, distinct from the Global Health Status/QoL score.asking.

Sleep quality was assessed using the Pittsburgh Sleep Quality Index (PSQI). Global PSQI scores range from 0 to 21 and were categorized as good sleep quality (0–5) or poor sleep quality (6–21). Anxiety was evaluated using the Generalized Anxiety Disorder-7 (GAD-7), with scores categorized as minimal (0–4), mild (5–9), moderate (10–14), or severe (15–21) anxiety.

Cognitive status was assessed using the Standardized Mini-Mental State Examination (SMMSE) and categorized as no cognitive impairment (24–30), mild cognitive impairment (18–23), or severe cognitive impairment (0–17). Nutritional status was evaluated using the Mini Nutritional Assessment–Short Form (MNA-SF) and classified as normal nutritional status (12–14), at risk of malnutrition (8–11), or malnutrition (0–7).

ECOG performance status was recorded at each assessment and categorized as 0–1 or 2–3 for analysis.

### 2.4. Treatment Tolerability and Tumor-Response Assessment

During the first 3 months of treatment, initial chemotherapy dose reduction, treatment-emergent dose reduction, treatment delay, Grade ≥ 3 treatment-related toxicity, and unplanned emergency department visits or hospitalizations were prospectively recorded. Treatment-related toxicities were graded according to the Common Terminology Criteria for Adverse Events (CTCAE), version 5.0. Initial dose reduction was defined as initiation of first-line chemotherapy at a reduced dose and was analyzed separately from treatment-emergent intolerance. Composite treatment intolerance was defined a priori as the occurrence of at least one of the following during the first 3 months: treatment-emergent dose reduction, treatment delay, Grade ≥ 3 treatment-related toxicity, or an unplanned emergency department visit or hospitalization. Initial dose reduction was not included in the composite endpoint. Initial dose reduction was considered a treatment decision made before treatment initiation, potentially reflecting baseline patient characteristics and clinical vulnerability rather than a treatment-emergent event. Furthermore, dose reductions and treatment delays occurring during treatment may reflect clinical management decisions and do not necessarily indicate biological intolerance. This composite endpoint was used to summarize treatment modifications and clinical events occurring during treatment, without assuming that its components shared the same clinical severity or underlying biological mechanism.

Tumor response was categorized as complete response (CR), partial response (PR), stable disease (SD), or progressive disease (PD) at the first radiologic response assessment performed as part of routine clinical care. The assessment was performed by the treating medical oncologist using computed tomography (CT) or positron emission tomography/computed tomography (PET/CT) images. Response classification was based on anatomical lesion measurements according to RECIST version 1.1; for PET/CT examinations, lesion measurements were derived from the CT component rather than metabolic uptake. Objective response rate (ORR) was defined as CR or PR and the disease control rate (DCR) as CR, PR, or SD. For analyses examining the association between tumor response and changes in quality of life, patients with CR, PR, or SD were classified as having disease control, whereas those with PD comprised the progressive-disease group. Because the initial radiologic response assessment was not performed at an identical fixed time point in every patient, these analyses were considered exploratory.

### 2.5. Study Endpoints

The primary endpoint was the change in EORTC QLQ-C30 Global Health Status/QoL from baseline to Month 3. Secondary outcomes included longitudinal changes in EORTC QLQ-C30 functional and symptom scales, PSQI, GAD-7, SMMSE, MNA-SF, ECOG performance status, and body weight, as well as treatment tolerability, composite treatment intolerance, and initial radiologic tumor response. A ≥10-point increase in the EORTC QLQ-C30 Global Health Status/QoL score from baseline to Month 3 was defined as a clinically meaningful improvement, whereas a ≥10-point decrease was defined as a clinically meaningful deterioration; changes between these thresholds were classified as stable quality of life [15]. This threshold was based on published evidence for interpreting changes in EORTC QLQ-C30 scores and was used as a pragmatic criterion for classifying individual changes rather than as a cutoff specifically validated in older patients with metastatic gastrointestinal cancer. Associations between initial tumor response and changes in quality of life, as well as PFS and OS analyses, were considered exploratory.

### 2.6. Statistical Analysis

All statistical analyses were performed using Python version 3.14 (Python Software Foundation, Beaverton, OR, USA). Data management and numerical computations were conducted using pandas (version 3.0.6; pandas Development Team), NumPy (version 2.5.3; NumPy Developers), and SciPy (version 1.18.1; SciPy Developers); regression, generalized estimating equation (GEE), and mixed-effects analyses were performed using statsmodels (version 0.15.0; statsmodels Developers). Bias-reduced Firth logistic regression and Firth penalized Cox regression were implemented using penalized-likelihood routines based on NumPy and SciPy optimization functions. All tests were two-sided, and a *p* value < 0.05 was considered statistically significant unless otherwise specified.

Continuous baseline variables were summarized as the median and interquartile range (IQR), whereas categorical variables were presented as frequencies and percentages. Longitudinal continuous outcomes were additionally summarized as the mean and standard deviation (SD). EORTC QLQ-C30 item-level responses outside the permissible range were treated as missing, and multi-item scales were calculated when at least half of the constituent items were available. No missing values were imputed.

Clinically meaningful change in Global Health Status/QoL at Month 3 was defined using a 10-point threshold. An increase of ≥10 points from baseline was classified as improvement, a decrease of ≥10 points as deterioration, and changes of <10 points in either direction as stable. Clinically meaningful QoL deterioration was additionally analyzed as a binary outcome.

The prespecified primary endpoint was the change in EORTC QLQ-C30 Global Health Status/QoL from baseline to Month 3. Longitudinal changes in functional and symptom domains of quality of life, sleep quality, anxiety, nutritional status, cognitive status, and performance status were evaluated as prespecified secondary outcomes. Analyses examining associations with tumor response, treatment intolerance, survival outcomes, and subgroup comparisons were considered exploratory. Findings from these exploratory analyses were interpreted as hypothesis-generating, particularly in the context of multiple comparisons. Longitudinal changes in Global Health Status/QoL, continuous QLQ-C30 functional and symptom scales, and body weight were evaluated using linear mixed-effects models, with assessment time (baseline, Month 1, and Month 3) entered as a categorical fixed effect and patient included as a random intercept. The overall effect of time was assessed using an omnibus test, and model-estimated changes from baseline to Month 1 and from baseline to Month 3 were reported with 95% confidence intervals (CIs). The baseline-to-Month 3 comparison constituted the primary contrast. Global Health Status/QoL was not included in multiplicity adjustment. For the 14 secondary EORTC QLQ-C30 functional and symptom domains, omnibus *p* values were adjusted using the Benjamini–Hochberg false discovery rate (FDR) procedure. No a priori sample-size calculation was performed. In a post hoc sensitivity-based power assessment using a paired-measures approximation, the available sample of 90 patients and the observed standard deviation of baseline-to-Month 3 within-patient Global Health Status/QoL changes (20.99 points) corresponded to an approximately 6.3-point detectable mean change at 80% power and a two-sided α of 0.05.

Repeated categorical patient-reported and geriatric measures were analyzed according to their measurement level. Overall longitudinal changes in binary PSQI and ECOG performance-status categories were assessed using Cochran’s Q test. When appropriate, pairwise comparisons between time points were performed using McNemar tests with Holm adjustment. GAD-7 and MNA-SF categories were evaluated using ordinal GEE models with patient-level clustering. Because SMMSE categories remained unchanged across the three assessment time points, no inferential longitudinal test was performed for SMMSE. Patient-level categorical transitions were additionally summarized descriptively as improved, unchanged, or worsened.

Treatment tolerability during the first 3 months included treatment-emergent chemotherapy dose reduction, treatment delay, Grade ≥ 3 treatment-related toxicity, and unplanned emergency department visit or hospitalization. Composite treatment intolerance was defined as the occurrence of at least one of these events. Initial chemotherapy dose reduction at treatment initiation was analyzed separately and was not included in the composite endpoint. Initial radiologic tumor response was assessed according to RECIST version 1.1 and classified as complete response (CR), partial response (PR), stable disease (SD), or progressive disease (PD). Objective response rate was defined as CR or PR and disease control as CR, PR, or SD.

Associations with clinically meaningful Month-3 QoL deterioration and treatment intolerance were first evaluated using univariable bias-reduced Firth logistic regression, with odds ratios (ORs), profile-likelihood 95% CIs, and *p* values reported. Candidate baseline variables included age, sex, ECOG performance status, body weight, baseline Global Health Status/QoL, PSQI, GAD-7, MNA-SF, SMMSE, chronic medication burden, fall history, living arrangement, comorbidities, primary tumor site, number of metastatic organs, and initial chemotherapy dose reduction. The multivariable model for QoL deterioration included age, ECOG performance status, and MNA-SF category because only 17 deterioration events were observed. Baseline Global Health Status/QoL was not included because the outcome was defined using change from the same baseline value. The multivariable treatment-intolerance model included age, ECOG performance status, MNA-SF category, baseline Global Health Status/QoL, and primary tumor site. Multivariable associations were estimated using Firth logistic regression and reported as adjusted ORs with 95% CIs.

The association between initial tumor response and longitudinal QoL was evaluated by comparing patients who achieved disease control (CR/PR/SD) with those who had PD. Changes from baseline to Month 3 in Global Health Status/QoL, physical functioning, fatigue, pain, and appetite loss were analyzed using Gaussian GEE models with patient-level clustering and terms for time, response group, and the time-by-response-group interaction. The interaction term represented the between-group difference in longitudinal change. *p* values across these five EORTC QLQ-C30 outcomes were adjusted using the Benjamini–Hochberg FDR procedure. Because initial radiologic response was not assessed at an identical fixed time point in every patient, these analyses were considered exploratory.

Exploratory sensitivity analyses examined whether longitudinal Global Health Status/QoL changes differed according to primary tumor site or first-line chemotherapy regimen using Gaussian GEE models containing time, subgroup, and time-by-subgroup interaction terms. Additional exploratory subgroup analyses assessed treatment intolerance and clinically meaningful QoL deterioration according to primary tumor site, chemotherapy regimen, receipt of targeted therapy, and receipt of immunotherapy using univariable Firth logistic regression. Because these analyses were exploratory and some subgroups were small, no multiplicity adjustment was applied, and effect estimates with 95% CIs were emphasized over isolated nominal *p* values.

Progression-free survival (PFS) was defined as the interval from initiation of first-line chemotherapy to radiologically documented disease progression or death from any cause, whichever occurred first. Overall survival (OS) was defined as the interval from initiation of first-line chemotherapy to death from any cause. Patients without an event were censored at the date of last clinical follow-up. Follow-up data were updated through 1 September 2026, which served as the data cutoff for the survival analyses. Median follow-up was estimated using the reverse Kaplan–Meier method. PFS and OS were estimated using the Kaplan–Meier method, with median survival, 95% CIs, and 6- and 12-month survival probabilities reported. Kaplan–Meier CIs were calculated using the log–log transformation.

Exploratory associations between baseline characteristics and PFS or OS were evaluated using univariable Cox proportional hazards regression and reported as hazard ratios (HRs) with 95% CIs. Because only 16 deaths occurred, no multivariable OS model was fitted. For PFS, with 35 events, a multivariable Firth penalized Cox model was fitted including age, ECOG performance status, MNA-SF category, SMMSE category, PSQI category, and primary tumor site. Survival analyses were considered exploratory, and no multiplicity adjustment was applied.

No last-observation-carried-forward or other single-value imputation procedure was used. Linear mixed-effects and GEE models used all available measurements under their respective likelihood- or estimating-equation frameworks. All 90 patients had baseline, Month 1, and Month 3 assessments for the principal longitudinal outcomes; scale-specific missingness arising from invalid or unavailable questionnaire responses was handled according to the scoring rules described above.

## 3. Results

A total of 90 older patients with de novo metastatic gastrointestinal cancer were prospectively enrolled, and all completed the baseline, Month 1, and Month 3 assessments (Supplementary Figure S1). The median age was 70.0 years (IQR, 66.0–72.8), and 61 patients (67.8%) were male. Most patients had an ECOG PS of 0–1 (85.6%). Colorectal cancer was the most common primary tumor site (52.2%), followed by esophagogastric (24.4%) and pancreaticobiliary cancers (23.3%). The median number of metastatic organs was 2 (IQR, 1–2), and the liver was the most frequent metastatic site (70.0%). Oxaliplatin–fluoropyrimidine-based chemotherapy was the predominant first-line regimen (78.9%), while targeted therapy and immunotherapy were administered as part of the first-line regimen in 46.7% and 13.3% of patients, respectively. The median baseline EORTC QLQ-C30 Global Health Status/QoL score was 66.7 (IQR, 52.1–83.3). At baseline, 31.1% of patients had poor sleep quality, 21.1% had moderate-to-severe anxiety, 26.7% had some degree of cognitive impairment, and 68.9% were either at risk of malnutrition or malnourished. Additional baseline demographic, clinical, treatment, and geriatric characteristics are summarized in Table 1.

The mean EORTC QLQ-C30 Global Health Status/QoL score was 68.1 (SD, 14.8) at baseline, 71.3 (SD, 16.0) at Month 1, and 71.0 (SD, 20.1) at Month 3. The model-estimated mean changes from baseline were +3.2 points (95% CI, 0.0 to 6.5) at Month 1 and +3.0 points (95% CI, −0.3 to 6.2) at Month 3, with no statistically significant overall effect of time (*p* = 0.091). The prespecified primary contrast from baseline to Month 3 also did not reach statistical significance (*p* = 0.071). Physical functioning and pain did not change significantly over time, whereas fatigue scores decreased by 6.5 points (95% CI, −11.0 to −2.1) at Month 1 and by 6.3 points (95% CI, −10.8 to −1.8) at Month 3 compared with baseline (overall *p* = 0.005). Appetite loss scores also decreased by 8.5 points (95% CI, −12.9 to −4.1) and 9.6 points (95% CI, −14.0 to −5.2), respectively (overall *p* < 0.001). The overall effect of time on body weight was not statistically significant (*p* = 0.079) (Table 2; Supplementary Figure S2).

Among categorical measures, the proportion of patients with poor sleep quality decreased from 31.1% at baseline to 23.3% at Month 1 and 21.1% at Month 3, with a significant overall change in PSQI categories over time (*p* = 0.011); however, none of the Holm-adjusted pairwise post-hoc comparisons reached statistical significance. MNA-SF categories changed significantly over time (*p* < 0.001), with the proportion of patients classified as having normal nutritional status increasing from 31.1% at baseline to 60.0% at Month 1 and 62.2% at Month 3. No significant longitudinal changes were observed in GAD-7 categories (*p* = 0.175) or ECOG PS (*p* = 0.232). SMMSE categories remained unchanged in all patients across the three assessment time points (Table 2 and Supplementary Figure S3).

**Figure 1:**
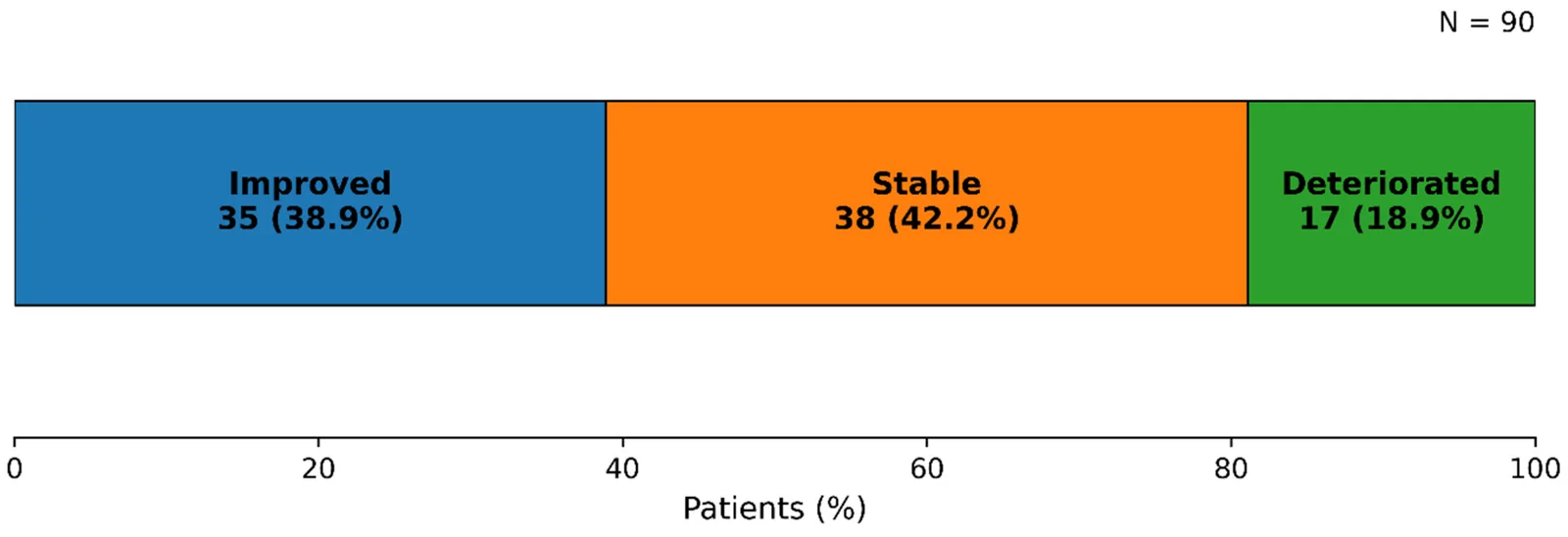
Clinically Meaningful Changes in EORTC QLQ-C30 Global Health Status/Quality of Life at Month 3. Abbreviations: EORTC QLQ-C30, European Organisation for Research and Treatment of Cancer Quality of Life Questionnaire Core 30; QoL, quality of life. Clinically meaningful change was defined according to the change in EORTC QLQ-C30 Global Health Status/QoL score from baseline to Month 3. Improvement was defined as an increase of ≥10 points, deterioration as a decrease of ≥10 points, and stability as a change of <10 points in either direction. Among 90 evaluable patients, 35 (38.9%) experienced clinically meaningful improvement, 38 (42.2%) remained stable, and 17 (18.9%) experienced clinically meaningful deterioration. Higher EORTC QLQ-C30 Global Health Status/QoL scores indicate better overall health status and quality of life.

When clinically meaningful change in EORTC QLQ-C30 Global Health Status/QoL from baseline to Month 3 was evaluated, 35 patients (38.9%) experienced an improvement of ≥10 points, 38 (42.2%) remained stable, and 17 (18.9%) experienced a deterioration of ≥10 points (Figure 1).

During the first 3 months of treatment, 45 patients (50.0%) started chemotherapy at a reduced dose. Treatment-emergent dose reduction occurred in 17 patients (18.9%), treatment delay in 38 (42.2%), Grade ≥ 3 treatment-related toxicity in 15 (16.7%), and an unplanned emergency department visit or hospitalization in 10 (11.1%). The composite treatment-intolerance endpoint, which excluded initial dose reduction, occurred in 42 patients (46.7%). At the initial radiologic response assessment, 6 patients (6.7%) achieved a complete response, 51 (56.7%) a partial response, 21 (23.3%) had stable disease, and 12 (13.3%) had progressive disease. The objective response rate was 63.3%, and the disease control rate was 86.7% (Table 3).

Clinically meaningful QoL deterioration at Month 3 occurred in 17 patients (18.9%). In multivariable analysis, age, ECOG PS, and baseline MNA-SF category were not independently associated with clinically meaningful QoL deterioration. Treatment intolerance during the first 3 months occurred in 42 patients (46.7%). In the treatment-intolerance model, colorectal primary tumor site was independently associated with lower odds of treatment intolerance compared with esophagogastric cancer (adjusted OR, 0.31; 95% CI, 0.10–0.88; *p* = 0.028). Age, ECOG PS, MNA-SF category, baseline EORTC QLQ-C30 Global Health Status/QoL score, and pancreaticobiliary primary tumor site were not significantly associated with treatment intolerance (Table 4).

**Figure 2:**
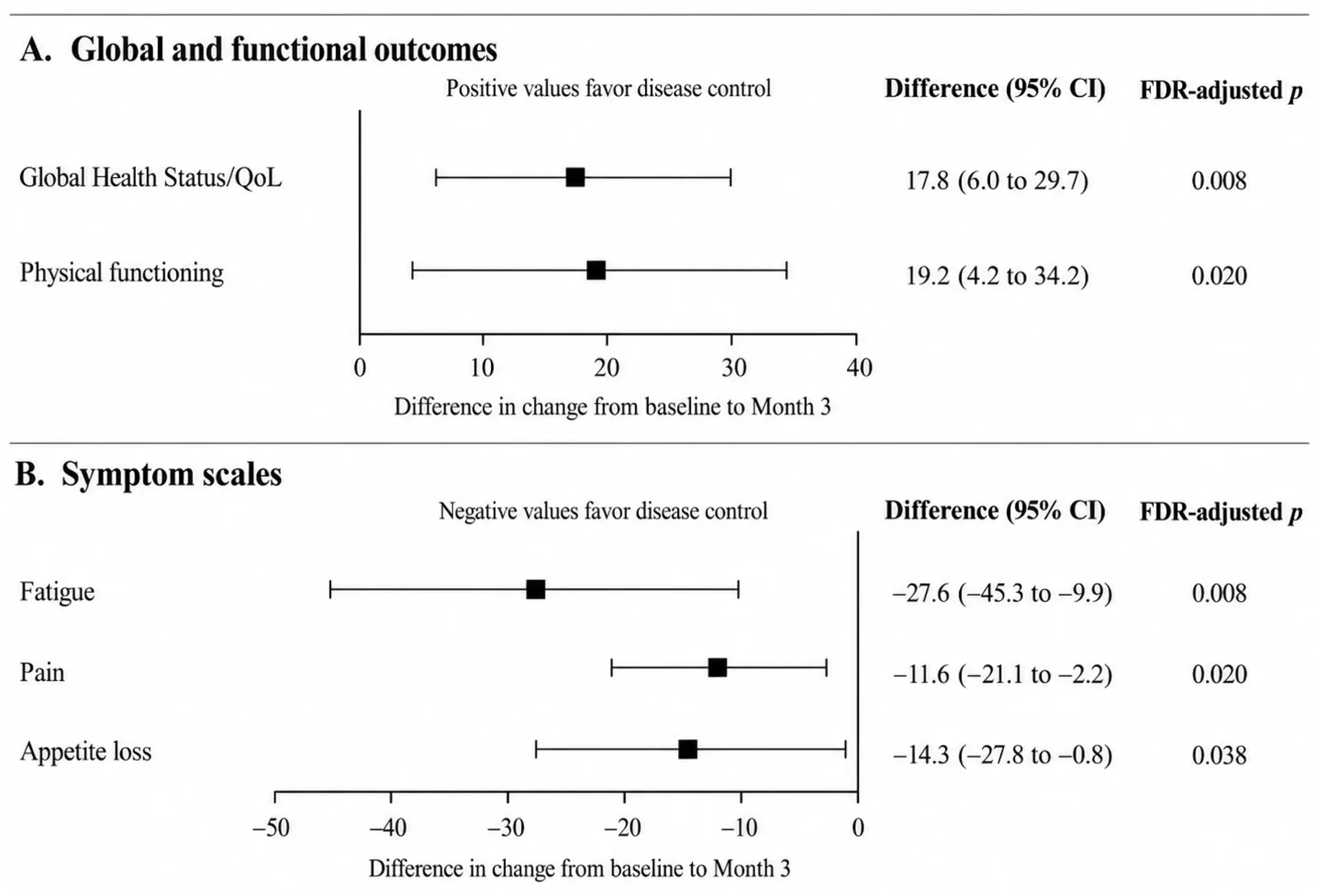
Differences in Changes in Quality of Life and Key Symptoms According to Initial Tumor Response. Abbreviations: CI, confidence interval; CR, complete response; EORTC QLQ-C30, European Organisation for Research and Treatment of Cancer Quality of Life Questionnaire Core 30; FDR, false discovery rate; PD, progressive disease; PR, partial response; QoL, quality of life; SD, stable disease. Forest plots show between-group differences in changes from baseline to Month 3 between patients who achieved disease control (CR/PR/SD) and those with progressive disease (PD). Panel (**A**) presents Global Health Status/QoL and physical functioning, for which positive values indicate greater improvement in the disease-control group. Panel (**B**) presents fatigue, pain, and appetite loss, for which negative values indicate greater symptom improvement in the disease-control group. Between-group differences and 95% CIs were estimated using Gaussian generalized estimating equations with patient-level clustering and terms for time, response group, and the time-by-response-group interaction. *p*-values were adjusted across the five prespecified EORTC QLQ-C30 outcomes using the Benjamini–Hochberg FDR procedure. Disease control was defined as complete response, partial response, or stable disease. Because tumor response represented the initial radiologic response and was not assessed at an identical fixed time point in every patient, these analyses should be interpreted as exploratory associations rather than causal effects.

Patients who achieved disease control at the initial radiologic assessment (CR/PR/SD; n = 78) had more favorable changes in quality of life and symptoms from baseline to Month 3 than those with progressive disease (n = 12). The EORTC QLQ-C30 Global Health Status/QoL score increased by a mean of 5.3 points in the disease-control group but decreased by 12.5 points in the progressive-disease group, yielding a between-group difference in change of 17.8 points (95% CI, 6.0 to 29.7; FDR-adjusted *p* = 0.008). Change in physical functioning was also more favorable in the disease-control group (between-group difference, 19.2 points; 95% CI, 4.2 to 34.2; FDR-adjusted *p* = 0.020). Similarly, changes in fatigue (difference, −27.6; 95% CI, −45.3 to −9.9; *p* = 0.008), pain (difference, −11.6; 95% CI, −21.1 to −2.2; *p* = 0.020), and appetite loss (difference, −14.3; 95% CI, −27.8 to −0.8; *p* = 0.038) favored patients with disease control. All reported *p*-values were FDR-adjusted (Supplementary Table S3; Figure 2).

In detailed analyses of the EORTC QLQ-C30 domains, several functional and symptom scales showed significant longitudinal changes after FDR adjustment for multiple comparisons. From baseline to Month 3, emotional functioning increased by 6.1 points (95% CI, 2.7 to 9.5; FDR-adjusted *p* = 0.005), and social functioning increased by 5.6 points (95% CI, 1.4 to 9.7; FDR-adjusted *p* = 0.015). Among symptom domains, fatigue decreased by 6.3 points (95% CI, −10.8 to −1.8; FDR-adjusted *p* = 0.018), insomnia by 4.8 points (95% CI, −8.7 to −0.9; FDR-adjusted *p* = 0.018), and appetite loss by 9.6 points (95% CI, −14.0 to −5.2; FDR-adjusted *p* < 0.001). Financial difficulties also decreased significantly (−4.8 points; 95% CI, −9.1 to −0.5; FDR-adjusted *p* = 0.036). No significant longitudinal changes were observed in the remaining EORTC QLQ-C30 functional or symptom domains after FDR adjustment (Supplementary Table S1).

At the individual level, 12 of 28 patients (42.9%) with poor sleep quality at baseline transitioned to the good-sleep-quality category by Month 3. From baseline to Month 3, PSQI category improved in 12 patients (13.3%) and worsened in 3 (3.3%). GAD-7 category improved in 17 patients (18.9%) and worsened in 6 (6.7%). The most prominent transitions were observed in nutritional status: among 57 patients who were at risk of malnutrition at baseline, 29 (50.9%) were classified as having normal nutritional status at Month 3, and overall MNA-SF category improved in 32 patients (35.6%). In contrast, ECOG PS category worsened in 9 patients (10.0%) and improved in 4 (4.4%). SMMSE categories remained unchanged in all patients from baseline to Month 3 (Supplementary Table S2).

In univariable analyses, none of the evaluated baseline factors was significantly associated with clinically meaningful QoL deterioration at Month 3. For treatment intolerance, coronary artery disease was associated with higher odds of intolerance (OR, 5.27; 95% CI, 1.38–28.97; *p* = 0.014). Compared with esophagogastric cancer, colorectal primary tumor site was associated with lower odds of treatment intolerance (OR, 0.30; 95% CI, 0.10–0.84; *p* = 0.022). In addition, each one-organ increase in the number of metastatic organs was associated with lower odds of treatment intolerance (OR, 0.53; 95% CI, 0.28–0.94; *p* = 0.030). No other evaluated demographic, clinical, patient-reported, geriatric, or treatment-related baseline factor was significantly associated with treatment intolerance (Supplementary Table S4).

The median follow-up, estimated using the reverse Kaplan–Meier method, was 6.8 months (95% CI, 5.7–7.8). A total of 35 patients (38.9%) experienced a PFS event, and the median PFS was 10.2 months (95% CI, 7.4–11.3). The estimated 6-month and 12-month PFS rates were 80.7% (95% CI, 70.3–87.7) and 26.8% (95% CI, 12.1–43.9), respectively. During follow-up, 16 deaths (17.8%) occurred. Median OS was not reached; the lower bound of the 95% CI was 12.2 months, with the upper bound not reached. The estimated 6-month and 12-month OS rates were 88.4% (95% CI, 78.6–93.8) and 71.2% (95% CI, 51.7–84.0), respectively (Supplementary Table S5; Supplementary Figure S4).

In univariable Cox regression analyses, being at risk of malnutrition or malnourished at baseline was associated with a higher risk of progression or death compared with normal nutritional status (HR, 2.71; 95% CI, 1.05–7.01; *p* = 0.040). Patients with any cognitive impairment had a higher risk of a PFS event than those without cognitive impairment (HR, 3.03; 95% CI, 1.23–7.69; *p* = 0.016). Similarly, poor sleep quality at baseline was associated with a higher risk of a PFS event (HR, 2.33; 95% CI, 1.00–5.26; *p* = 0.049). None of the other evaluated baseline factors was significantly associated with PFS. No baseline factor showed a statistically significant association with OS (Supplementary Table S6).

In multivariable Firth penalized Cox regression analysis, being at risk of malnutrition or malnourished at baseline was independently associated with a higher risk of progression or death compared with normal nutritional status (adjusted HR, 3.58; 95% CI, 1.34–9.55; *p* = 0.011). Similarly, poor sleep quality at baseline was independently associated with a higher risk of a PFS event (adjusted HR, 3.30; 95% CI, 1.33–8.15; *p* = 0.010). Primary tumor site was significantly associated with PFS overall (overall *p* = 0.014); however, neither the colorectal nor pancreaticobiliary category reached statistical significance individually compared with esophagogastric cancer. Age, ECOG PS, and SMMSE were not significantly associated with PFS in the multivariable model (Supplementary Table S7).

The change in EORTC QLQ-C30 Global Health Status/QoL from baseline to Month 3 did not differ significantly according to primary tumor site (*p* for interaction = 0.596). Similarly, longitudinal QoL changes did not differ across first-line chemotherapy regimens (*p* for interaction = 0.531). Treatment intolerance occurred in 68.2% of patients with esophagogastric cancer, 38.3% of those with colorectal cancer, and 42.9% of those with pancreaticobiliary cancer; colorectal primary tumor site was associated with lower odds of treatment intolerance compared with esophagogastric cancer (OR, 0.30; 95% CI, 0.10–0.84; *p* = 0.022). Receipt of targeted therapy was associated with lower odds of treatment intolerance (OR, 0.37; 95% CI, 0.15–0.85; *p* = 0.018), whereas receipt of immunotherapy was associated with higher odds (OR, 3.69; 95% CI, 1.07–15.69; *p* = 0.038). For clinically meaningful QoL deterioration, gemcitabine-based chemotherapy was associated with higher odds of deterioration compared with oxaliplatin–fluoropyrimidine-based therapy (OR, 3.86; 95% CI, 1.05–13.81; *p* = 0.043). No other subgroup comparisons were statistically significant (Supplementary Table S8). No multiplicity adjustment was applied to these exploratory subgroup analyses.

## 4. Discussion

The principal finding of this study was that mean global quality of life showed no statistically significant change during the first 3 months of first-line systemic therapy in older patients with de novo metastatic gastrointestinal cancers. The mean change from baseline to Month 3 was +3.0 points (95% CI, −0.3 to 6.2), indicating no clear evidence of a substantial mean deterioration. Studies focusing exclusively on metastatic colorectal cancer provide relevant context for our findings; however, their populations are not directly comparable to our heterogeneous cohort, which also included esophagogastric and pancreaticobiliary cancers. In the NORDIC9 trial, quality of life was generally preserved during treatment in older and vulnerable patients with metastatic colorectal cancer [1]. Similarly, a prospective real-world study of patients with newly diagnosed metastatic colorectal cancer receiving first-line palliative chemotherapy showed that older patients did not experience greater deterioration in health-related quality of life than younger patients [16]. However, the absence of a statistically significant change in mean quality-of-life scores may not fully capture the heterogeneity of individual patient experiences. In a prospective study by Decoster et al. involving older patients with cancer, quality of life improved in some patients and deteriorated in others over a 3-month follow-up period [17]. Likewise, in our cohort, despite the absence of a statistically significant change in mean global quality of life, clinically meaningful improvement and deterioration were both observed at the individual level, underscoring the importance of examining individual changes alongside group-level averages.

Favorable changes were observed in fatigue, appetite loss, and MNA-SF–based nutritional risk categories. MNA-SF categories changed significantly over time (overall *p* < 0.001), with the proportion of patients classified as having normal nutritional status increasing from 31.1% at baseline to 62.2% at Month 3. Previous studies have reported varying patterns of nutritional outcomes during anticancer treatment. In a recent randomized trial involving patients aged ≥65 years with gastric cancer receiving neoadjuvant chemotherapy, high-protein oral nutritional supplementation was associated with significant improvement in MNA scores and a reduction in the risk of malnutrition after 8 weeks [18]. In contrast, in a study by Bıçaklı et al. involving patients aged ≥65 years with gastrointestinal cancers, the prevalence of malnutrition increased from 37.9% to 46.4% after one cycle of chemotherapy (*p* = 0.001) [19]. These contrasting findings suggest that nutritional risk may follow heterogeneous trajectories during treatment in older patients with gastrointestinal cancers. Importantly, the MNA-SF is a nutritional screening tool rather than a direct measure of dietary intake or body composition. The observed changes in our cohort may have been influenced by reduced appetite loss, disease control, or supportive care provided during routine clinical follow-up, and regression to the mean cannot be excluded. Therefore, changes in MNA-SF categories should not be interpreted as definitive evidence of restored nutritional status or attributed directly to systemic therapy.

In our study, patients who achieved disease control at the initial radiologic assessment experienced significantly more favorable changes in global quality of life, physical functioning, fatigue, pain, and appetite loss than those with progressive disease. This finding is consistent with previous studies suggesting that tumor response may be associated with changes in quality of life during treatment. In a prospective study by Shin et al. involving patients with advanced gastrointestinal tumors, improvement in global quality of life and/or emotional functioning was observed among patients who achieved an objective response or stable disease [20]. Similarly, Kato et al. reported that global quality of life improved significantly in patients with gastrointestinal cancer who achieved a partial response to chemotherapy, whereas overall quality-of-life scores deteriorated in those with progressive disease [21]. However, the assessment of radiologic response at variable post-baseline time points limits the interpretation of the observed association. Differences in disease course and patient characteristics may also have influenced changes in quality of life. Therefore, these findings should be regarded as exploratory evidence of a potential association rather than evidence that disease control caused improvements in quality of life.

In our exploratory survival analysis, baseline risk of malnutrition/malnutrition and poor sleep quality remained associated with shorter progression-free survival after multivariable adjustment for age, ECOG performance status, cognitive status, and primary tumor site. With respect to nutritional status, a systematic review evaluating the prognostic value of the Mini Nutritional Assessment in patients with cancer reported that malnutrition or risk of malnutrition was associated with shorter progression-free survival or time to progression in several of the included studies [22]. Similarly, in a prospective study of patients with advanced non-small-cell lung cancer, poor sleep quality assessed using the Pittsburgh Sleep Quality Index was independently associated with shorter progression-free survival [23]. These findings suggest that baseline nutritional status and sleep quality may provide additional information potentially associated with clinical outcomes in older patients with cancer. However, these measures may also be influenced by factors such as disease burden, frailty, symptom severity, and overall clinical condition. Although the multivariable model adjusted for selected clinical characteristics, potential residual confounding related to treatment regimen, metastatic disease burden, and other indicators of disease severity cannot be excluded. Given the limited number of PFS events, these associations require cautious interpretation and validation in larger prospective cohorts.

Several specific quality-of-life domains showed statistically significant changes during treatment. By Month 3, emotional and social functioning had improved, whereas insomnia, fatigue, and appetite loss had decreased, with these changes remaining significant after adjustment for multiple comparisons. Nevertheless, given the magnitude of the observed changes, statistical significance may not necessarily indicate clinically meaningful improvement. Therefore, the clinical relevance of changes in individual domains should be interpreted cautiously, considering established domain-specific thresholds for clinical meaningfulness where available. These findings suggest that global quality-of-life scores alone may not fully capture domain-specific changes occurring during treatment. Similarly, in a longitudinal study by Esbensen et al. involving patients aged ≥65 years with newly diagnosed cancer, emotional functioning improved significantly over 6 months, while quality of life remained stable in approximately 70% of patients [24]. These observations suggest that assessing functional and symptom-specific domains alongside global quality of life may be useful for evaluating the patient experience during treatment in older adults with cancer.

Beyond patient-reported outcomes, treatment tolerability was also clinically relevant in this older population. Half of the patients initiated chemotherapy at a reduced dose, and 46.7% experienced at least one treatment-intolerance event during the first 3 months. These findings highlight the importance of individualizing treatment intensity and supportive care needs in older patients. Consistent with this, the GAIN trial demonstrated that geriatric assessment–guided multidisciplinary interventions can reduce severe treatment-related toxicity in older adults with cancer [7]. In our cohort, colorectal primary tumor site was associated with lower odds of treatment intolerance compared with esophagogastric cancer; however, given the heterogeneity of tumor types and treatment regimens, this finding should be considered exploratory. Nevertheless, the composite treatment-intolerance endpoint included events that differed in their clinical significance and potential underlying causes. In particular, treatment-emergent dose reductions and treatment delays may reflect clinician-directed treatment decisions rather than biological intolerance.

Therefore, this endpoint should be interpreted as a broad measure of treatment modifications and clinical events rather than as a homogeneous indicator of biological intolerance.

The main strengths of this study include its prospective longitudinal design, complete assessments at baseline, Month 1, and Month 3, and the concurrent evaluation of quality of life together with sleep quality, anxiety, nutritional status, cognitive status, and performance status. Nevertheless, several limitations should be considered. The single-center design and relatively limited sample size may restrict the generalizability of the findings. Furthermore, the absence of an a priori sample-size calculation aligned with the primary endpoint represents an additional limitation. As indicated by the post hoc sensitivity analysis, the available sample may have had limited statistical power to detect smaller changes in global quality of life. For future prospective studies targeting a 5-point mean within-patient change in EORTC QLQ-C30 Global Health Status/QoL from baseline to Month 3, an estimated 141 evaluable patients would be required to achieve 80% power at a two-sided α of 0.05, assuming a standard deviation of paired changes of 20.99 points as observed in this cohort. This illustrative estimate should be refined according to the clinically justified target difference and anticipated attrition. Therefore, given that the study was not designed to establish non-inferiority or equivalence, the nonsignificant primary result should not be interpreted as definitive evidence that quality of life was preserved. The absence of an untreated or alternative-treatment comparison group also limits the extent to which the observed changes in quality of life can be attributed to systemic therapy. In addition, the inclusion of different gastrointestinal primary tumors and chemotherapy regimens, as well as the concomitant use of targeted therapy or immunotherapy in some patients, introduced clinical heterogeneity. Although exploratory analyses did not identify statistically significant differences in longitudinal quality-of-life changes according to primary tumor site or chemotherapy regimen, the limited sample size restricts the interpretation of these subgroup findings. Because treatment regimens were selected according to clinical indications rather than randomly assigned, confounding by indication may have influenced longitudinal quality-of-life, treatment-tolerability, nutritional, and survival outcomes. The 3-month follow-up period for patient-reported and geriatric outcomes limits the assessment of longer-term changes. The use of categorical classifications for selected geriatric and patient-reported measures may have limited the detection of potential changes within categories. Therefore, particularly for cognitive status, unchanged categorical classifications may not reflect stability in underlying scores or function. Although assessments were conducted according to standardized procedures and without knowledge of previous assessment results, the use of the same investigator for repeated evaluations may have introduced interviewer-related measurement bias.

Survival analyses were also exploratory and were based on only 35 PFS events and 16 deaths; therefore, these findings should be interpreted cautiously. Given the limited number of PFS events relative to the number of model parameters, the multivariable estimates may be unstable and susceptible to overfitting despite the use of Firth penalization. Furthermore, the large number of exploratory comparisons relative to the sample size may have increased the likelihood of chance findings. Results from analyses without adjustment for multiple comparisons should therefore be interpreted as hypothesis-generating rather than confirmatory. Finally, because the initial radiologic response was not assessed at an identical fixed time point in all patients and the study was observational in design, caution is warranted when interpreting the association between tumor response and changes in quality of life as reflecting a causal effect.

## 5. Conclusions

In this single-center, prospective observational study of 90 older patients with de novo metastatic gastrointestinal cancers, mean global quality of life showed no statistically significant change during the first 3 months of first-line systemic therapy. Favorable changes were observed in some symptom and functional domains. More favorable quality-of-life trajectories were associated with disease control at the initial radiologic assessment, while baseline nutritional impairment and poor sleep quality were associated with shorter progression-free survival in exploratory analyses. However, these findings should be interpreted cautiously given the observational design, limited sample size, and short assessment period. Further prospective studies with larger cohorts and longer follow-up are needed to validate these observations.

## Figures and Tables

**Table 1:** Baseline Demographic, Clinical, Tumor, Treatment, and Geriatric Characteristics of the Study Population.

Characteristic	Overall Cohort (*N* = 90), *n* (%)
Demographic characteristics	
Age, years, median (IQR)	70.0 (66.0–72.8)
Sex	
Female	29 (32.2)
Male	61 (67.8)
Education level	
Primary school	62 (68.9)
Middle school	8 (8.9)
High school	12 (13.3)
University	8 (8.9)
Baseline clinical characteristics	
Body weight, kg, median (IQR)	68.0 (58.0–79.8)
BMI, kg/m^2^, median (IQR)	25.4 (22.5–28.5)
ECOG PS	
0–1	77 (85.6)
2–3	13 (14.4)
Primary tumor site	
Esophagogastric	22 (24.4)
Colorectal	47 (52.2)
Pancreaticobiliary	21 (23.3)
Metastatic disease characteristics	
Number of metastatic organs, median (IQR)	2 (1–2)
Liver metastasis	63 (70.0)
Lung metastasis	28 (31.1)
Peritoneal metastasis	25 (27.8)
Bone metastasis	8 (8.9)
Lymph-node metastasis	37 (41.1)
Primary tumor resection	27 (30.0)
First-line treatment characteristics	
Oxaliplatin–fluoropyrimidine-based chemotherapy	71 (78.9)
Oxaliplatin–irinotecan–fluoropyrimidine-based chemotherapy	7 (7.8)
Gemcitabine-based chemotherapy	12 (13.3)
Targeted therapy as part of the first-line regimen	42 (46.7)
Immunotherapy as part of the first-line regimen	12 (13.3)
Baseline quality-of-life, patient-reported, and geriatric measures	
EORTC QLQ-C30 Global Health Status/QoL, median (IQR)	66.7 (52.1–83.3)
PSQI	
Good sleep quality (0–5)	62 (68.9)
Poor sleep quality (6–21)	28 (31.1)
GAD-7	
Minimal anxiety (0–4)	41 (45.6)
Mild anxiety (5–9)	30 (33.3)
Moderate anxiety (10–14)	15 (16.7)
Severe anxiety (15–21)	4 (4.4)
SMMSE	
No cognitive impairment (24–30)	66 (73.3)
Mild cognitive impairment (18–23)	22 (24.4)
Severe cognitive impairment (0–17)	2 (2.2)
MNA-SF	
Normal nutritional status (12–14)	28 (31.1)
At risk of malnutrition (8–11)	57 (63.3)
Malnutrition (0–7)	5 (5.6)
Other geriatric characteristics	
Number of chronic medications	
0	35 (38.9)
1	20 (22.2)
2	4 (4.4)
3	16 (17.8)
≥4	15 (16.7)
Falls during the previous 6 months	
None	82 (91.1)
≥1	8 (8.9)
Living arrangement	
Alone	6 (6.7)
With spouse/family	83 (92.2)
With caregiver	1 (1.1)
Comorbidities	
Diabetes mellitus	30 (33.3)
Hypertension	29 (32.2)
Coronary artery disease	11 (12.2)
COPD	4 (4.4)
Other comorbidity	14 (15.6)

Abbreviations: BMI, body mass index; COPD, chronic obstructive pulmonary disease; ECOG PS, Eastern Cooperative Oncology Group performance status; EORTC QLQ-C30, European Organisation for Research and Treatment of Cancer Quality of Life Questionnaire Core 30; GAD-7, Generalized Anxiety Disorder-7; IQR, interquartile range; SMMSE, Standardized Mini-Mental State Examination; MNA-SF, Mini Nutritional Assessment Short Form; PSQI, Pittsburgh Sleep Quality Index; QoL, quality of life. Data are presented as n (%) unless otherwise specified. Continuous variables are presented as median (IQR). BMI was calculated from baseline body weight and height as weight in kilograms divided by height in meters squared. EORTC QLQ-C30 Global Health Status/QoL was transformed to the standard 0–100 scale, with higher scores indicating better overall health status and quality of life. Metastatic sites and comorbidities were not mutually exclusive. Targeted therapy and immunotherapy indicate agents administered as part of the first-line systemic treatment regimen. Percentages may not total 100% because of rounding.

**Table 2:** Longitudinal Changes in Quality of Life, Patient-Reported Outcomes, and Geriatric Measures During First-Line Chemotherapy.

**A. Continuous measures**							
**Measure**	**Baseline (** ***N*** ** = 90)**	**Month 1 (** ***N*** ** = 90)**	**Month 3 (** ***N*** ** = 90)**	**Change, Month 1 vs. Baseline (95% CI)**		**Change, Month 3 vs. Baseline (95% CI)**	**Overall ** ***p*** **-value**
EORTC QLQ-C30 Global Health Status/QoL	68.1 (14.8)	71.3 (16.0)	71.0 (20.1)	+3.2 (0.0 to 6.5)		+3.0 (−0.3 to 6.2)	0.091
Physical functioning	72.4 (22.6)	75.9 (21.3)	74.7 (25.1)	+3.4 (−0.2 to 7.1)		+2.2 (−1.4 to 5.9)	0.178
Fatigue	35.1 (25.1)	28.5 (22.6)	28.8 (27.8)	−6.5 (−11.0 to −2.1)		−6.3 (−10.8 to −1.8)	0.005
Pain	16.1 (18.1)	15.7 (18.8)	15.7 (19.4)	−0.4 (−2.8 to 2.1)		−0.4 (−2.8 to 2.1)	0.943
Appetite loss	34.4 (30.6)	25.9 (28.6)	24.8 (30.2)	−8.5 (−12.9 to −4.1)		−9.6 (−14.0 to −5.2)	<0.001
Body weight, kg	69.7 (14.7)	70.0 (14.6)	70.3 (14.9)	+0.2 (−0.3 to 0.7)		+0.6 (0.1 to 1.1)	0.079
**B. Categorical patient-reported and geriatric measures**							
**Measure**	**Baseline (** ***N*** ** = 90)**	**Month 1 (** ***N*** ** = 90)**			**Month 3 (** ***N*** ** = 90)**		**Overall ** ***p*** **-value**
PSQI							0.011 †
Good sleep quality	62 (68.9)	69 (76.7)			71 (78.9)		
Poor sleep quality	28 (31.1)	21 (23.3)			19 (21.1)		
GAD-7							0.175 ‡
Minimal anxiety	41 (45.6)	39 (43.3)			42 (46.7)		
Mild anxiety	30 (33.3)	40 (44.4)			39 (43.3)		
Moderate anxiety	15 (16.7)	11 (12.2)			8 (8.9)		
Severe anxiety	4 (4.4)	0 (0.0)			1 (1.1)		
MNA-SF							<0.001 ‡
Normal nutritional status	28 (31.1)	54 (60.0)			56 (62.2)		
At risk of malnutrition	57 (63.3)	32 (35.6)			30 (33.3)		
Malnutrition	5 (5.6)	4 (4.4)			4 (4.4)		
SMMSE							—
No cognitive impairment	66 (73.3)	66 (73.3)			66 (73.3)		
Mild cognitive impairment	22 (24.4)	22 (24.4)			22 (24.4)		
Severe cognitive impairment	2 (2.2)	2 (2.2)			2 (2.2)		
ECOG PS							0.232 †
0–1	77 (85.6)	75 (83.3)			72 (80.0)		
2–3	13 (14.4)	15 (16.7)			18 (20.0)		

Abbreviations: CI, confidence interval; ECOG PS, Eastern Cooperative Oncology Group performance status; EORTC QLQ-C30, European Organisation for Research and Treatment of Cancer Quality of Life Questionnaire Core 30; GAD-7, Generalized Anxiety Disorder-7; SMMSE, Standardized Mini-Mental State Examination; MNA-SF, Mini Nutritional Assessment–Short Form; PSQI, Pittsburgh Sleep Quality Index; QoL, quality of life. Continuous data are presented as mean (SD), and categorical data as n (%). Longitudinal changes in EORTC QLQ-C30 scores and body weight were evaluated using linear mixed-effects models with time (baseline, Month 1, and Month 3) entered as a categorical fixed effect and patient as a random intercept. Reported changes represent model-estimated mean differences from baseline with 95% CIs. For each 95% CI, the first value represents the lower confidence limit (LCL), and the second value represents the upper confidence limit (UCL). The prespecified primary contrast was the change in EORTC QLQ-C30 Global Health Status/QoL from baseline to Month 3 (mean difference, +3.0 points; 95% CI, −0.3 to 6.2; *p* = 0.071). Higher EORTC QLQ-C30 Global Health Status/QoL and functional scores indicate better functioning, whereas higher symptom scores indicate greater symptom burden. Out-of-range EORTC item-level responses were treated as missing before scoring; multi-item scales were calculated when at least half of the constituent items were available according to EORTC scoring principles. All five EORTC measures shown in this table remained evaluable for all 90 patients at all three time points. This refers to the availability of calculable scale scores and does not necessarily imply complete item-level responses. Evaluable sample sizes for the remaining EORTC QLQ-C30 domains are provided in Supplementary Table S1. † Overall changes in binary repeated measures (PSQI and ECOG PS) were assessed using Cochran’s Q test. For PSQI, post-hoc pairwise McNemar tests with Holm correction yielded adjusted *p*-values of 0.185 for baseline versus Month 1, 0.105 for baseline versus Month 3, and 0.500 for Month 1 versus Month 3. ‡ Longitudinal changes in ordinal outcomes (GAD-7 and MNA-SF) were assessed using ordinal generalized estimating equations with patient-level clustering. SMMSE categories remained unchanged in all patients across the three assessment time points; therefore, no inferential test for longitudinal change was performed. Two-sided *p*-values < 0.05 were considered statistically significant.

**Table 3:** Treatment Tolerability During the First 3 Months and Initial Tumor Response to First-Line Chemotherapy.

Outcome	Overall Cohort (*N* = 90), *n* (%)
Treatment tolerability during the first 3 months	
Initial chemotherapy dose reduction	45 (50.0)
Dose reduction during the first 3 months	17 (18.9)
Treatment delay during the first 3 months	38 (42.2)
Grade ≥ 3 treatment-related toxicity	15 (16.7)
Unplanned emergency department visit or hospitalization	10 (11.1)
Composite treatment intolerance	
Any treatment intolerance event	42 (46.7)
No treatment intolerance event	48 (53.3)
Initial tumor response	
Complete response (CR)	6 (6.7)
Partial response (PR)	51 (56.7)
Stable disease (SD)	21 (23.3)
Progressive disease (PD)	12 (13.3)
Objective response rate (CR + PR)	57 (63.3)
Disease control rate (CR + PR + SD)	78 (86.7)

Abbreviations: CR, complete response; PD, progressive disease; PR, partial response; SD, stable disease. Data are presented as n (%). All 90 patients had available data for the treatment-tolerability variables and initial tumor-response assessment. Initial chemotherapy dose reduction refers to a reduced chemotherapy dose prescribed at the initiation of first-line treatment and was analyzed separately from treatment-emergent intolerance. Composite treatment intolerance was defined a priori as the occurrence of at least one of the following during the first 3 months of treatment: treatment-emergent chemotherapy dose reduction, treatment delay, Grade ≥ 3 treatment-related toxicity, or an unplanned emergency department visit or hospitalization. Initial dose reduction was not included in the composite endpoint. ORR was defined as CR or PR, and DCR was defined as CR, PR, or SD. Individual components of the composite endpoint were not mutually exclusive; therefore, their percentages should not be summed.

**Table 4:** Multivariable Factors Associated With Clinically Meaningful Quality-of-Life Deterioration and Treatment Intolerance.

Factor	Adjusted OR (95% CI)	*p *-Value
A. Clinically meaningful QoL deterioration at Month 3 (17/90, 18.9%)		
Age, per 5-year increase	1.28 (0.71–2.30)	0.405
ECOG PS 2–3 vs. 0–1	0.72 (0.12–3.02)	0.669
MNA-SF at risk of malnutrition/malnourished vs. normal	0.82 (0.28–2.55)	0.725
B. Treatment intolerance during the first 3 months (42/90, 46.7%)		
Age, per 5-year increase	1.05 (0.63–1.76)	0.848
ECOG PS 2–3 vs. 0–1	1.50 (0.41–5.69)	0.535
MNA-SF at risk of malnutrition/malnourished vs. normal	0.79 (0.29–2.13)	0.634
Baseline EORTC QLQ-C30 Global Health Status/QoL, per 10-point increase	0.84 (0.59–1.17)	0.300
Primary tumor site		
Esophagogastric	Reference	—
Colorectal	0.31 (0.10–0.88)	0.028
Pancreaticobiliary	0.35 (0.09–1.21)	0.097

Abbreviations: CI, confidence interval; ECOG PS, Eastern Cooperative Oncology Group performance status; EORTC QLQ-C30, European Organisation for Research and Treatment of Cancer Quality of Life Questionnaire Core 30; MNA-SF, Mini Nutritional Assessment–Short Form; OR, odds ratio; QoL, quality of life. Clinically meaningful QoL deterioration was defined a priori as a decrease of ≥10 points in the EORTC QLQ-C30 Global Health Status/QoL score from baseline to Month 3. Treatment intolerance was defined as the occurrence of at least one of the following during the first 3 months of first-line chemotherapy: treatment-emergent dose reduction, treatment delay, Grade ≥ 3 treatment-related toxicity, or an unplanned emergency department visit or hospitalization. Initial chemotherapy dose reduction was not included in the composite treatment-intolerance endpoint. Multivariable associations were estimated using bias-reduced Firth logistic regression, and results are reported as adjusted ORs with profile-likelihood 95% CIs. For each 95% CI, the first value represents the lower confidence limit (LCL), and the second value represents the upper confidence limit (UCL). Because only 17 QoL-deterioration events occurred, Model A was deliberately restricted to three clinically prespecified baseline factors to reduce overfitting. Baseline QoL was not included in Model A because the outcome itself was defined using change from the baseline QoL value, thereby avoiding mathematical coupling and regression-to-the-mean bias. Model B additionally included baseline Global Health Status/QoL and primary tumor site because 42 treatment-intolerance events permitted a broader prespecified adjustment set. MNA-SF was dichotomized as normal nutritional status versus at risk of malnutrition/malnourished because only five patients had established malnutrition. All 90 patients had complete data for the variables included in these models. Two-sided *p*-values < 0.05 were considered statistically significant.

## Data Availability

This study is a prospective clinical investigation involving patient-level clinical data, quality-of-life assessments, psychological, evaluations, and other patient-reported outcomes. To protect patient privacy, maintain the confidentiality of personal health information, and comply with ethical principles in clinical research, de-identified data may be made available by the corresponding author upon reasonable request.

## Supplementary Materials

The following supporting information can be downloaded at: https://www.mdpi.com/article/10.3390/healthcare14203458/s1, Supplementary material includes Supplementary Table S1, Detailed Longitudinal Changes in EORTC QLQ-C30 Functional and Symptom Scales During First-Line Chemotherapy; Supplementary Table S2, Longitudinal Transitions in Patient-Reported and Geriatric Categories During First-Line Chemotherapy; Supplementary Table S3, Association Between Initial Tumor Response and Changes in Quality of Life and Key Symptoms During First-Line Chemotherapy; Supplementary Table S4, Univariable Factors Associated With Clinically Meaningful Quality-of-Life Deterioration and Treatment Intolerance; Supplementary Table S5, Progression-Free and Overall Survival Outcomes of the Study Cohort; Supplementary Table S6, Exploratory Univariable Cox Regression Analyses for Progression-Free and Overall Survival; Supplementary Table S7, Multivariable Cox Regression Analysis for Progression-Free Survival; Supplementary Table S8, Exploratory Sensitivity Analyses of Quality-of-Life Changes and Treatment Intolerance Across Tumor and Treatment Subgroups; Supplementary Figure S1, Study Flow Diagram; Supplementary Figure S2, Longitudinal Changes in EORTC QLQ-C30 Global Health Status/Quality of Life During First-Line Chemotherapy; Supplementary Figure S3, Longitudinal Changes in Patient-Reported and Geriatric Measures During First-Line Chemotherapy; and Supplementary Figure S4, Kaplan–Meier Curves for Progression-Free and Overall Survival.
