# Supplementary material for: Quality of Life and Patient-Reported Outcomes During First-Line Chemotherapy in Older Patients with De Novo Metastatic Gastrointestinal Cancers: A Prospective Longitudinal Study
Source: Healthcare (Basel). 2026 Oct 15;14(20):3458. doi: 10.3390/healthcare14203458 (PMC13704573; doi:10.3390/healthcare14203458)
Supplement: Supplementary file 1 [file healthcare-14-03458-s001.zip › healthcare-4603719-supplementary.pdf]

Supplementary Table S1. Detailed Longitudinal Changes in EORTC QLQ-C30 Functional and Symptom Scales During First-Line Chemotherapy

EORTC QLQ-C30 scale	Baseline, mean (SD)	Month 1, mean (SD)	Month 3, mean (SD)	Change, Month 1 vs Baseline (95% CI)	Change, Month 3 vs Baseline (95% CI)	Overall p-value	FDR-adjusted p-value
**Global Health Status/QoL**	68.1 (14.8)	71.3 (16.0)	71.0 (20.1)	+3.2 (0.0 to 6.5)	+3.0 (−0.3 to 6.2)	0.091	—
**Functional scales**							
Physical functioning	72.4 (22.6)	75.9 (21.3)	74.7 (25.1)	+3.4 (−0.2 to 7.1)	+2.2 (−1.4 to 5.9)	0.178	0.277
Role functioning	65.7 (26.1)	71.5 (23.3)	70.0 (27.6)	+5.7 (0.9 to 10.6)	+4.3 (−0.6 to 9.1)	0.053	0.102
Emotional functioning	72.0 (28.5)	77.2 (21.9)	78.1 (23.2)	+5.2 (1.8 to 8.6)	+6.1 (2.7 to 9.5)	**<0.001**	**0.005**
Cognitive functioning	92.4 (15.4)	92.4 (13.5)	93.0 (12.7)	+0.0 (−1.8 to 1.8)	+0.6 (−1.2 to 2.3)	0.780	0.840
Social functioning	70.9 (24.8)	77.6 (22.4)	76.5 (24.3)	+6.7 (2.5 to 10.8)	+5.6 (1.4 to 9.7)	**0.003**	**0.015**
**Symptom scales/items**							
Fatigue	35.1 (25.1)	28.5 (22.6)	28.8 (27.8)	−6.5 (−11.0 to −2.1)	−6.3 (−10.8 to −1.8)	**0.005**	**0.018**
Nausea/vomiting	6.7 (15.2)	8.0 (15.6)	7.2 (14.4)	+1.3 (−0.7 to 3.3)	+0.6 (−1.5 to 2.6)	0.448	0.570
Pain	16.1 (18.1)	15.7 (18.8)	15.7 (19.4)	−0.4 (−2.8 to 2.1)	−0.4 (−2.8 to 2.1)	0.943	0.943
Dyspnea*	5.3 (12.3)	7.2 (15.5)	7.1 (16.3)	+1.2 (−0.7 to 3.2)	+1.2 (−0.7 to 3.2)	0.353	0.494
Insomnia	21.5 (26.6)	15.6 (23.5)	16.7 (26.6)	−5.9 (−9.8 to −2.0)	−4.8 (−8.7 to −0.9)	**0.006**	**0.018**
Appetite loss	34.4 (30.6)	25.9 (28.6)	24.8 (30.2)	−8.5 (−12.9 to −4.1)	−9.6 (−14.0 to −5.2)	**<0.001**	**<0.001**
Constipation	25.2 (22.0)	23.3 (22.0)	22.6 (23.3)	−1.9 (−4.0 to 0.3)	−2.6 (−4.8 to −0.4)	0.058	0.102
Diarrhea	11.1 (18.7)	10.0 (18.3)	10.4 (20.4)	−1.1 (−3.7 to 1.5)	−0.7 (−3.3 to 1.9)	0.694	0.809
Financial difficulties	28.9 (25.1)	23.0 (22.7)	24.1 (24.5)	−5.9 (−10.2 to −1.6)	−4.8 (−9.1 to −0.5)	**0.016**	**0.036**

Abbreviations: CI, confidence interval; EORTC QLQ-C30, European Organisation for Research and Treatment of Cancer Quality of Life Questionnaire Core 30; FDR, false discovery rate; QoL, quality of life; SD, standard deviation.

EORTC QLQ-C30 scores were calculated according to standard scoring principles and transformed to a 0–100 scale. Higher scores on Global Health Status/QoL and functional scales indicate better health status or functioning, whereas higher scores on symptom scales/items indicate greater symptom burden. Descriptive values are presented as mean (SD). Longitudinal changes were evaluated using linear mixed-effects models with assessment time (baseline, Month 1, and Month 3) entered as a categorical fixed effect and patient as a random intercept. Changes represent model-estimated mean differences from baseline with 95% CIs. The overall p-value represents the omnibus effect of time across the three assessments. Global Health Status/QoL was the prespecified primary quality-of-life endpoint and was therefore not included in multiplicity adjustment. For the 14 secondary EORTC QLQ-C30 functional and symptom domains, overall p-values were adjusted using the Benjamini–Hochberg FDR procedure. Out-of-range item responses were treated as missing before scoring; for multi-item scales, a score was calculated when at least half of the constituent items were available. All scales were evaluable in 90 patients at each assessment except dyspnea, for which evaluable sample sizes were 88 at baseline, 88 at Month 1, and 89 at Month 3. Two-sided p-values <0.05 were considered statistically significant.

Supplementary Table S2. Longitudinal Transitions in Patient-Reported and Geriatric Categories During First-Line Chemotherapy

Panel A. Transitions From Baseline to Month 1

Measure	Baseline category, n	Month 1 category	n (%)
**PSQI**	Good sleep quality, n=62	Good sleep quality	59 (95.2)
		Poor sleep quality	3 (4.8)
	Poor sleep quality, n=28	Good sleep quality	10 (35.7)
		Poor sleep quality	18 (64.3)
**GAD-7**	Minimal anxiety, n=41	Minimal	38 (92.7)
		Mild	3 (7.3)
	Mild anxiety, n=30	Minimal	1 (3.3)
		Mild	26 (86.7)
		Moderate	3 (10.0)
	Moderate anxiety, n=15	Mild	11 (73.3)
		Moderate	4 (26.7)

Measure	Baseline category, n	Month 1 category	n (%)
	Severe anxiety, n=4	Moderate	4 (100.0)
**SMMSE**	No cognitive impairment, n=66	No cognitive impairment	66 (100.0)
	Mild cognitive impairment, n=22	Mild cognitive impairment	22 (100.0)
	Severe cognitive impairment, n=2	Severe cognitive impairment	2 (100.0)
**MNA-SF**	Normal nutritional status, n=28	Normal	26 (92.9)
		At risk of malnutrition	2 (7.1)
	At risk of malnutrition, n=57	Normal	28 (49.1)
		At risk of malnutrition	27 (47.4)
		Malnutrition	2 (3.5)
	Malnutrition, n=5	At risk of malnutrition	3 (60.0)
		Malnutrition	2 (40.0)
**ECOG PS**	0–1, n=77	0–1	72 (93.5)
		2–3	5 (6.5)
	2–3, n=13	0–1	3 (23.1)
		2–3	10 (76.9)

Panel B. Transitions From Baseline to Month 3

Measure	Baseline category, n	Month 3 category	n (%)
**PSQI**	Good sleep quality, n=62	Good sleep quality	59 (95.2)
		Poor sleep quality	3 (4.8)
	Poor sleep quality, n=28	Good sleep quality	12 (42.9)
		Poor sleep quality	16 (57.1)
**GAD-7**	Minimal anxiety, n=41	Minimal	38 (92.7)
		Mild	1 (2.4)
		Moderate	2 (4.9)
	Mild anxiety, n=30	Minimal	2 (6.7)
		Mild	25 (83.3)
		Moderate	2 (6.7)
		Severe	1 (3.3)
	Moderate anxiety, n=15	Minimal	2 (13.3)
		Mild	9 (60.0)
		Moderate	4 (26.7)
	Severe anxiety, n=4	Mild	4 (100.0)
**SMMSE**	No cognitive impairment, n=66	No cognitive impairment	66 (100.0)
	Mild cognitive impairment, n=22	Mild cognitive impairment	22 (100.0)
	Severe cognitive impairment, n=2	Severe cognitive impairment	2 (100.0)
**MNA-SF**	Normal nutritional status, n=28	Normal	26 (92.9)
		At risk of malnutrition	2 (7.1)
	At risk of malnutrition, n=57	Normal	29 (50.9)
		At risk of malnutrition	26 (45.6)
		Malnutrition	2 (3.5)
	Malnutrition, n=5	Normal	1 (20.0)
		At risk of malnutrition	2 (40.0)
		Malnutrition	2 (40.0)
**ECOG PS**	0–1, n=77	0–1	68 (88.3)
		2–3	9 (11.7)
	2–3, n=13	0–1	4 (30.8)
		2–3	9 (69.2)

Panel C. Direction of Categorical Change

Measure	Time interval	Improved, n (%)	Unchanged, n (%)	Worsened, n (%)
**PSQI**	Baseline → Month 1	10 (11.1)	77 (85.6)	3 (3.3)
	Baseline → Month 3	12 (13.3)	75 (83.3)	3 (3.3)

Measure	Time interval	Improved, n (%)	Unchanged, n (%)	Worsened, n (%)
**GAD-7**	Baseline → Month 1	16 (17.8)	68 (75.6)	6 (6.7)
	Baseline → Month 3	17 (18.9)	67 (74.4)	6 (6.7)
**SMMSE**	Baseline → Month 1	0 (0.0)	90 (100.0)	0 (0.0)
	Baseline → Month 3	0 (0.0)	90 (100.0)	0 (0.0)
**MNA-SF**	Baseline → Month 1	31 (34.4)	55 (61.1)	4 (4.4)
	Baseline → Month 3	**32 (35.6)**	54 (60.0)	4 (4.4)
**ECOG PS**	Baseline → Month 1	3 (3.3)	82 (91.1)	5 (5.6)
	Baseline → Month 3	4 (4.4)	77 (85.6)	**9 (10.0)**

Abbreviations: ECOG PS, Eastern Cooperative Oncology Group performance status; GAD-7, Generalized Anxiety Disorder-7; SMMSE, Standardized Mini-Mental State Examination; MNA-SF, Mini Nutritional Assessment–Short Form; PSQI, Pittsburgh Sleep Quality Index.

Transition percentages in Panels A and B are calculated within each baseline category (row percentages). Panel C summarizes the direction of change among all 90 patients. For all measures shown, movement to a numerically lower category represents improvement and movement to a numerically higher category represents worsening. PSQI categories were good versus poor sleep quality; GAD-7 categories were minimal, mild, moderate, and severe anxiety; SMMSE categories were no, mild, and severe cognitive impairment; MNA-SF categories were normal nutritional status, at risk of malnutrition, and malnutrition; and ECOG PS categories were 0–1 versus 2–3. All 90 patients had available categorical assessments at baseline, Month 1, and Month 3. SMMSE categories remained unchanged in every patient across all three assessments; therefore, no inferential test for longitudinal SMMSE category change was performed. Inferential longitudinal analyses for these categorical outcomes are reported in Table 2.

Supplementary Table S3. Association Between Initial Tumor Response and Changes in Quality of Life and Key Symptoms During First-Line Chemotherapy

Outcome, change from baseline to Month 3	Disease control (CR/PR/SD) (n = 78), mean (SD)	Progressive disease (n = 12), mean (SD)	Between-group difference in change (95% CI)	FDR-adjusted p-value
**Global Health Status/QoL**	+5.3 (20.2)	−12.5 (20.3)	**+17.8 (6.0 to 29.7)**	**0.008**
**Physical functioning**	+4.8 (22.0)	−14.4 (26.2)	**+19.2 (4.2 to 34.2)**	**0.020**
**Fatigue**	−10.0 (26.2)	+17.6 (30.9)	**−27.6 (−45.3 to −9.9)**	**0.008**
**Pain**	−1.9 (13.7)	+9.7 (16.6)	**−11.6 (−21.1 to −2.2)**	**0.020**
**Appetite loss**	−11.5 (27.3)	+2.8 (22.3)	**−14.3 (−27.8 to −0.8)**	**0.038**

Abbreviations: CI, confidence interval; CR, complete response; EORTC QLQ-C30, European Organisation for Research and Treatment of Cancer Quality of Life Questionnaire Core 30; FDR, false discovery rate; PD, progressive disease; PR, partial response; QoL, quality of life; SD, stable disease.

Data in the response groups are presented as mean (SD) changes from baseline to Month 3. Disease control was defined as complete response, partial response, or stable disease, whereas progressive disease was categorized as no disease control. Between-group differences in longitudinal change were estimated using Gaussian generalized estimating equations with patient-level clustering and terms for time, response group, and the time-by-response-group interaction. The reported effect corresponds to the interaction term representing the difference in change from baseline to Month 3 between the disease-control and progressive-disease groups. P-values were adjusted across the five prespecified EORTC QLQ-C30 outcomes using the Benjamini–Hochberg false discovery rate procedure. For Global Health Status/QoL and physical functioning, positive changes indicate improvement; for fatigue, pain, and appetite loss, negative changes indicate improvement. All 90 patients had evaluable baseline and Month-3 data for the outcomes shown. Because tumor response was assessed as the initial radiologic response rather than at an identical fixed time point in every patient, these analyses should be interpreted as exploratory associations and not as evidence of causality.

Supplementary Table S4. Univariable Factors Associated With Clinically Meaningful Quality-of-Life Deterioration and Treatment Intolerance

Baseline factor	QoL deterioration at Month 3, OR (95% CI)	p-value	Treatment intolerance, OR (95% CI)	p-value
**Demographic and clinical factors**				
Age, per 5-year increase	1.24 (0.70–2.13)	0.449	1.30 (0.83–2.08)	0.250
Male vs female	1.12 (0.38–3.68)	0.835	1.11 (0.46–2.69)	0.816
ECOG PS 2–3 vs 0–1	0.88 (0.16–3.40)	0.860	1.95 (0.62–6.60)	0.255
Body weight, per 10-kg increase	1.02 (0.71–1.44)	0.931	0.94 (0.71–1.24)	0.658

Baseline factor	QoL deterioration at Month 3, OR (95% CI)	p-value	Treatment intolerance, OR (95% CI)	p-value
Baseline Global Health Status/QoL, per 10-point increase	1.24 (0.88–1.78)	0.218	0.83 (0.62–1.10)	0.195
**Baseline patient-reported and geriatric measures**				
Poor vs good sleep quality (PSQI)	0.46 (0.11–1.49)	0.203	0.53 (0.21–1.30)	0.167
Moderate/severe vs minimal/mild anxiety (GAD-7)	0.27 (0.03–1.22)	0.095	1.34 (0.50–3.68)	0.560
At risk of malnutrition/malnourished vs normal MNA-SF	0.77 (0.27–2.39)	0.643	1.01 (0.42–2.46)	0.980
Any cognitive impairment vs no impairment (SMMSE)	0.59 (0.14–1.93)	0.399	1.49 (0.59–3.81)	0.394
≥4 vs 0–3 chronic medications	0.37 (0.04–1.71)	0.226	2.56 (0.85–8.45)	0.095
≥1 fall during previous 6 months vs none	1.67 (0.29–7.35)	0.530	0.40 (0.07–1.69)	0.221
Living alone vs not living alone	2.49 (0.40–12.41)	0.297	4.64 (0.87–46.72)	0.073
**Comorbidities**				
Diabetes mellitus, yes vs no	0.60 (0.17–1.82)	0.375	1.22 (0.51–2.91)	0.655
Hypertension, yes vs no	0.89 (0.27–2.62)	0.835	1.64 (0.68–3.99)	0.269
Coronary artery disease, yes vs no	0.55 (0.06–2.62)	0.488	**5.27 (1.38–28.97)**	**0.014**
COPD, yes vs no	0.44 (0.003–4.45)	0.550	1.15 (0.17–7.75)	0.881
Other comorbidity, yes vs no	0.79 (0.14–3.04)	0.753	1.61 (0.53–5.11)	0.400
**Tumor and treatment characteristics**				
**Primary tumor site**				
Esophagogastric	Reference	—	Reference	—
Colorectal	0.76 (0.21–2.98)	0.681	**0.30 (0.10–0.84)**	**0.022**
Pancreaticobiliary	1.72 (0.44–7.28)	0.435	0.37 (0.10–1.21)	0.101
Number of metastatic organs, per 1-organ increase	1.26 (0.63–2.46)	0.499	**0.53 (0.28–0.94)**	**0.030**
Initial chemotherapy dose reduction, yes vs no	2.75 (0.95–8.91)	0.063	1.19 (0.52–2.72)	0.676

Abbreviations: CI, confidence interval; COPD, chronic obstructive pulmonary disease; ECOG PS, Eastern Cooperative Oncology Group performance status; EORTC QLQ-C30, European Organisation for Research and Treatment of Cancer Quality of Life Questionnaire Core 30; GAD-7, Generalized Anxiety Disorder-7; SMMSE, Standardized Mini-Mental State Examination; MNA-SF, Mini Nutritional Assessment–Short Form; OR, odds ratio; PSQI, Pittsburgh Sleep Quality Index; QoL, quality of life.

Clinically meaningful QoL deterioration was defined as a decrease of ≥10 points in the EORTC QLQ-C30 Global Health Status/QoL score from baseline to Month 3 and occurred in 17 of 90 patients (18.9%). Treatment intolerance was defined as the occurrence of at least one treatment-emergent dose reduction, treatment delay, Grade ≥3 treatment-related toxicity, or unplanned emergency department visit/hospitalization during the first 3 months and occurred in 42 of 90 patients (46.7%). Each baseline factor was evaluated separately using univariable bias-reduced Firth logistic regression. ORs are reported with profile-likelihood 95% CIs. Age, body weight, baseline Global Health Status/QoL, and number of metastatic organs were modeled as continuous variables using the increments indicated in the table. GAD-7 was dichotomized as minimal/mild versus moderate/severe anxiety; MNA-SF as normal versus at risk of malnutrition/malnourished; SMMSE as no impairment versus any cognitive impairment; and chronic medication burden as 0–3 versus ≥4 medications. "Not living alone" included patients living with a spouse/family or caregiver. Initial chemotherapy dose reduction refers to a reduced dose prescribed at treatment initiation and was not itself included in the composite treatment-intolerance endpoint. Baseline Global Health Status/QoL is presented in the univariable QoL-deterioration analysis for completeness but was not entered into the multivariable QoL-deterioration model because the outcome was defined using change from that same baseline value. No multiplicity adjustment was applied to these exploratory univariable analyses; effect estimates and 95% CIs should therefore be emphasized over isolated p-values. All analyses included 90 patients.

Supplementary Table S5. Progression-Free and Overall Survival Outcomes of the Study Cohort

Survival outcome	Estimate	95% CI
**Median follow-up, months**	**6.8**	**5.7–7.8**
**Progression-free survival**		
PFS events, n (%)	35 (38.9)	—
Median PFS, months	**10.2**	**7.4–11.3**
6-month PFS rate, %	**80.7**	**70.3–87.7**
12-month PFS rate, %	**26.8**	**12.1–43.9**

Survival outcome	Estimate	95% CI
**Overall survival**		
Deaths, n (%)	16 (17.8)	—
Median OS, months	**Not reached**	**12.2–NR**
6-month OS rate, %	**88.4**	**78.6–93.8**
12-month OS rate, %	**71.2**	**51.7–84.0**

Abbreviations: CI, confidence interval; NR, not reached; OS, overall survival; PFS, progression-free survival.

Median follow-up was estimated using the reverse Kaplan–Meier method. PFS was defined as the interval from initiation of first-line chemotherapy to radiologically documented disease progression or death from any cause, whichever occurred first; patients without a PFS event were censored at the date of last clinical follow-up. OS was defined as the interval from initiation of first-line chemotherapy to death from any cause; patients alive at the last known follow-up were censored on that date. Median PFS and OS and landmark survival probabilities were estimated using the Kaplan–Meier method with 95% CIs. The median OS was not reached at the time of data cutoff; its 95% CI was therefore bounded below at 12.2 months with the upper limit not reached. At 12 months, 6 patients remained at risk for PFS and 12 for OS; accordingly, 12-month estimates should be interpreted with their corresponding confidence intervals.

Supplementary Table S6. Exploratory Univariable Cox Regression Analyses for Progression-Free and Overall Survival

Baseline factor	PFS HR (95% CI)	p-value	OS HR (95% CI)	p-value
**Demographic and clinical factors**				
Age, per 5-year increase	1.07 (0.76–1.50)	0.698	0.84 (0.49–1.47)	0.548
Male vs female	0.97 (0.48–1.95)	0.924	1.60 (0.52–4.97)	0.416
ECOG PS 2–3 vs 0–1	1.79 (0.78–4.11)	0.172	1.61 (0.46–5.67)	0.460
**Baseline patient-reported and geriatric measures**				
MNA-SF at risk of malnutrition/malnourished vs normal	**2.71 (1.05–7.01)**	**0.040**	2.85 (0.64–12.63)	0.168
Any cognitive impairment vs no impairment (SMMSE)	**3.03 (1.23–7.69)**	**0.016**	1.45 (0.47–4.55)	0.517
Moderate/severe vs minimal/mild anxiety (GAD-7)	1.07 (0.47–2.46)	0.872	1.51 (0.48–4.74)	0.481
Poor vs good sleep quality (PSQI)	**2.33 (1.00–5.26)**	**0.049**	2.33 (0.66–8.33)	0.190
Baseline Global Health Status/QoL, per 10-point increase	1.01 (0.79–1.28)	0.946	1.03 (0.72–1.47)	0.888
**Tumor and treatment characteristics**				
**Primary tumor site**				
Esophagogastric	Reference	—	Reference	—
Colorectal	0.55 (0.23–1.33)	0.183	0.48 (0.13–1.80)	0.278
Pancreaticobiliary	2.40 (0.97–5.93)	0.058	2.04 (0.59–7.08)	0.261
Number of metastatic organs, per 1-organ increase	1.41 (0.92–2.17)	0.115	1.23 (0.64–2.36)	0.529
Initial chemotherapy dose reduction, yes vs no	1.37 (0.70–2.66)	0.361	2.31 (0.80–6.67)	0.121

Abbreviations: CI, confidence interval; ECOG PS, Eastern Cooperative Oncology Group performance status; EORTC QLQ-C30, European Organisation for Research and Treatment of Cancer Quality of Life Questionnaire Core 30; GAD-7, Generalized Anxiety Disorder-7; HR, hazard ratio; SMMSE, Standardized Mini-Mental State Examination; MNA-SF, Mini Nutritional Assessment–Short Form; OS, overall survival; PFS, progression-free survival; PSQI, Pittsburgh Sleep Quality Index; QoL, quality of life.

PFS was defined as the interval from initiation of first-line chemotherapy to radiologically documented disease progression or death from any cause, whichever occurred first. OS was defined as the interval from initiation of first-line chemotherapy to death from any cause. Each baseline factor was evaluated separately using an univariable Cox proportional hazards model. Analyses included 90 patients, with 35 PFS events and 16 deaths. Age and baseline Global Health Status/QoL were modeled per 5-year and 10-point increments, respectively, and number of metastatic organs per 1-organ increment. GAD-7 was dichotomized as minimal/mild versus moderate/severe anxiety; MNA-SF as normal versus at risk of malnutrition/malnourished; SMMSE as no cognitive impairment versus any cognitive impairment; and PSQI as good versus poor sleep quality. Esophagogastric cancer served as the reference category for primary tumor site. No multiplicity adjustment was applied because these survival analyses were prespecified as exploratory. Given the limited number of OS events, particularly only 16 deaths, no multivariable Cox model was fitted, and HR estimates, especially those with wide CIs, should be interpreted cautiously.

Supplementary Table S7. Multivariable Cox Regression Analysis for Progression-Free Survival

Baseline factor	Adjusted HR (95% CI)	p-value
Age, per 5-year increase	1.27 (0.85–1.89)	0.246

Baseline factor	Adjusted HR (95% CI)	p-value
ECOG PS 2–3 vs 0–1	1.97 (0.74–5.23)	0.175
**MNA-SF at risk of malnutrition/malnourished vs normal**	**3.58 (1.34–9.55)**	**0.011**
Any cognitive impairment vs no cognitive impairment (SMMSE)	2.32 (0.88–6.14)	0.091
**Poor vs good sleep quality (PSQI)**	**3.30 (1.33–8.15)**	**0.010**
**Primary tumor site**		**0.014***
Esophagogastric	Reference	—
Colorectal	0.56 (0.21–1.49)	0.249
Pancreaticobiliary	2.28 (0.89–5.86)	0.087

Abbreviations: CI, confidence interval; ECOG PS, Eastern Cooperative Oncology Group performance status; HR, hazard ratio; SMMSE, Standardized Mini-Mental State Examination; MNA-SF, Mini Nutritional Assessment–Short Form; PFS, progression-free survival; PSQI, Pittsburgh Sleep Quality Index.

Multivariable associations with PFS were estimated using Firth penalized Cox proportional hazards regression to reduce small-sample and sparse-event bias. The analysis included all 90 patients and 35 PFS events. PFS was defined as the interval from initiation of first-line chemotherapy to radiologically documented disease progression or death from any cause, whichever occurred first. Age was modeled continuously per 5-year increase. ECOG PS was categorized as 0–1 versus 2–3; MNA-SF as normal nutritional status versus at risk of malnutrition/malnourished; SMMSE as any cognitive impairment versus no cognitive impairment; and PSQI as poor versus good sleep quality, with the contrasts displayed in the table indicating the direction of the reported HRs. Esophagogastric cancer served as the reference category for primary tumor site. HRs are presented with 95% Wald CIs. *The p-value for primary tumor site represents the overall 2-degree-of-freedom test for the categorical variable. Because this survival analysis was exploratory and based on 35 PFS events, effect estimates and CIs should be emphasized, and the results require cautious interpretation and external validation.

Supplementary Table S8. Exploratory Sensitivity Analyses of Quality-of-Life Changes and Treatment Intolerance Across Tumor and Treatment Subgroups

Panel A. Longitudinal Change in Global Health Status/Quality of Life According to Primary Tumor Site

Primary tumor site	N	Baseline Global QoL, mean (SD)	Month 3 Global QoL, mean (SD)	Model-estimated change (95% CI)	P for interaction
Esophagogastric	22	68.6 (11.5)	75.4 (20.3)	+6.8 (−2.5 to 16.1)	
Colorectal	47	70.0 (16.7)	72.2 (20.3)	+2.1 (−4.0 to 8.2)	
Pancreaticobiliary	21	63.1 (12.5)	63.9 (18.5)	+0.8 (−6.7 to 8.2)	**0.596**

Panel B. Longitudinal Change in Global Health Status/Quality of Life According to First-Line Chemotherapy Regimen

First-line chemotherapy regimen	N	Baseline Global QoL, mean (SD)	Month 3 Global QoL, mean (SD)	Model-estimated change (95% CI)	P for interaction
Oxaliplatin–fluoropyrimidine-based	71	69.0 (15.1)	73.0 (20.1)	+4.0 (−1.0 to 9.0)	
Oxaliplatin–irinotecan–fluoropyrimidine-based	7	65.5 (10.1)	65.5 (10.1)	0.0 (−6.6 to 6.6)	
Gemcitabine-based	12	63.9 (15.6)	62.5 (22.6)	−1.4 (−13.2 to 10.5)	**0.531**

Panel C. Treatment Intolerance According to Tumor and Treatment Subgroups

Subgroup	Treatment intolerance, n/N (%)	OR (95% CI)	p-value
**Primary tumor site**			
Esophagogastric	15/22 (68.2)	Reference	—
Colorectal	18/47 (38.3)	**0.30 (0.10–0.84)**	**0.022**
Pancreaticobiliary	9/21 (42.9)	0.37 (0.10–1.21)	0.101
**First-line chemotherapy regimen**			
Oxaliplatin–fluoropyrimidine-based	33/71 (46.5)	Reference	—
Oxaliplatin–irinotecan–fluoropyrimidine-based	4/7 (57.1)	1.48 (0.34–7.04)	0.601
Gemcitabine-based	5/12 (41.7)	0.84 (0.24–2.77)	0.778
**Targeted therapy**			
No	28/48 (58.3)	Reference	—
Yes	14/42 (33.3)	**0.37 (0.15–0.85)**	**0.018**
**Immunotherapy**			
No	33/78 (42.3)	Reference	—
Yes	9/12 (75.0)	**3.69 (1.07–15.69)**	**0.038**

Panel D. Clinically Meaningful Quality-of-Life Deterioration According to Tumor and Treatment Subgroups

Subgroup	QoL deterioration, n/N (%)	OR (95% CI)	p-value
**Primary tumor site**			
Esophagogastric	4/22 (18.2)	Reference	—
Colorectal	7/47 (14.9)	0.76 (0.21–2.98)	0.681
Pancreaticobiliary	6/21 (28.6)	1.72 (0.44–7.28)	0.435
**First-line chemotherapy regimen**			
Oxaliplatin–fluoropyrimidine-based	11/71 (15.5)	Reference	—
Oxaliplatin–irinotecan–fluoropyrimidine-based	1/7 (14.3)	1.21 (0.12–6.63)	0.843
Gemcitabine-based	5/12 (41.7)	**3.86 (1.05–13.81)**	**0.043**
**Targeted therapy**			
No	12/48 (25.0)	Reference	—
Yes	5/42 (11.9)	0.43 (0.13–1.24)	0.120
**Immunotherapy**			
No	13/78 (16.7)	Reference	—
Yes	4/12 (33.3)	2.57 (0.66–9.07)	0.165

Abbreviations: CI, confidence interval; EORTC QLQ-C30, European Organisation for Research and Treatment of Cancer Quality of Life Questionnaire Core 30; OR, odds ratio; QoL, quality of life; SD, standard deviation.

These analyses were prespecified as exploratory sensitivity analyses to assess whether the principal findings were materially influenced by heterogeneity in primary tumor site or systemic treatment. In Panels A and B, longitudinal changes in EORTC QLQ-C30 Global Health Status/QoL from baseline to Month 3 were analyzed using Gaussian generalized estimating equations with patient-level clustering and terms for time, subgroup, and the time-by-subgroup interaction. Model-estimated changes represent within-subgroup changes from baseline to Month 3 with 95% CIs; the interaction p-value tests whether the magnitude of longitudinal QoL change differed across subgroup levels. Treatment intolerance in Panel C was defined as the occurrence of at least one treatment-emergent dose reduction, treatment delay, Grade ≥3 treatment-related toxicity, or unplanned emergency department visit/hospitalization during the first 3 months; initial chemotherapy dose reduction was not included in this composite endpoint. Clinically meaningful QoL deterioration in Panel D was defined as a decrease of ≥10 points in EORTC QLQ-C30 Global Health Status/QoL from baseline to Month 3. Associations in Panels C and D were estimated using univariable bias-reduced Firth logistic regression, with ORs and profile-likelihood 95% CIs. No multiplicity adjustment was applied because all subgroup analyses were exploratory; therefore, isolated nominal p-values should be interpreted cautiously, particularly in small treatment subgroups. All 90 patients had evaluable data for the analyses shown.

Supplementary Figure S1. Study Flow Diagram

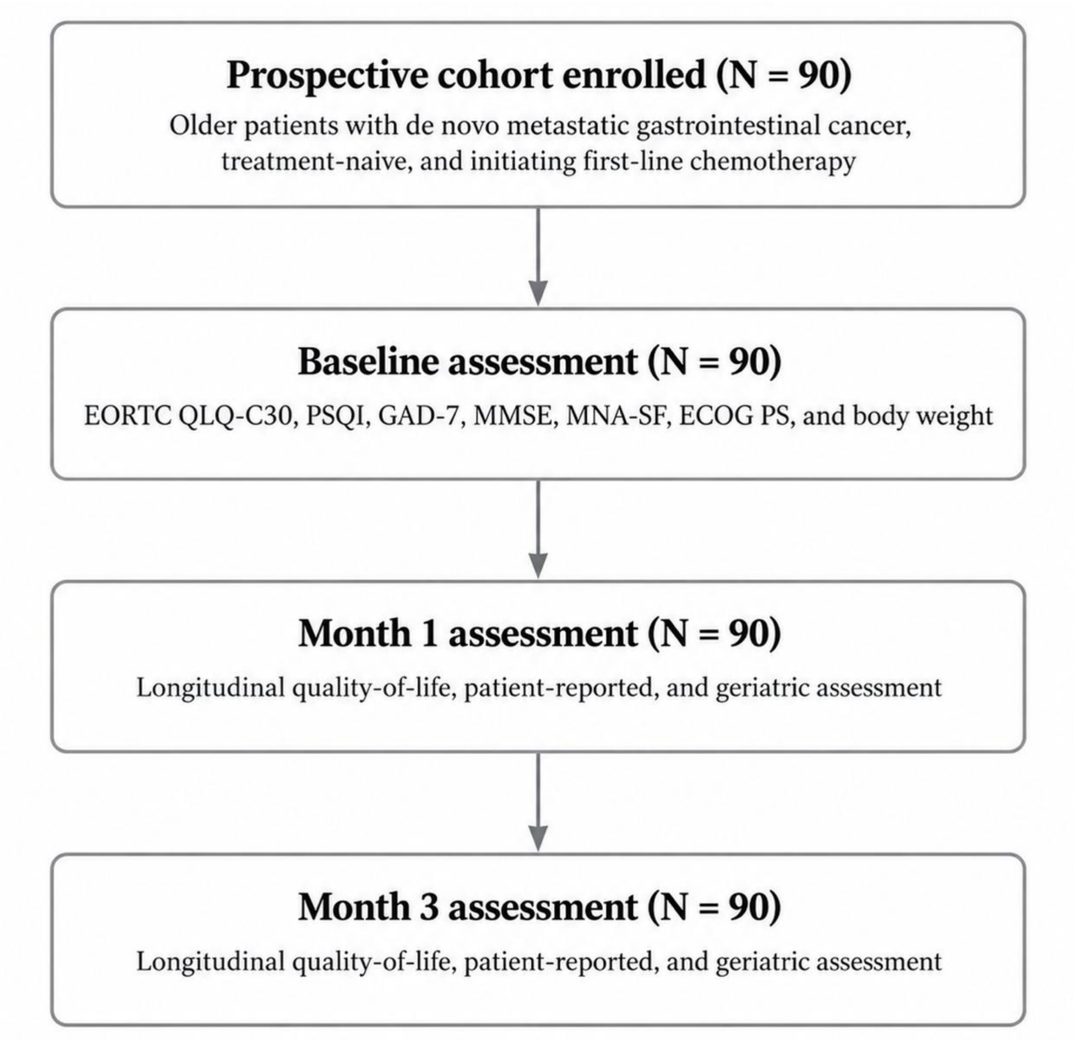

Abbreviations: ECOG PS, Eastern Cooperative Oncology Group performance status; EORTC QLQ-C30, European Organisation for Research and Treatment of Cancer Quality of Life Questionnaire Core 30; GAD-7, Generalized Anxiety Disorder-7; SMMSE, Standardized Mini-Mental State Examination; MNA-SF, Mini Nutritional Assessment–Short Form; PSQI, Pittsburgh Sleep Quality Index.

The study included 90 older patients with de novo metastatic gastrointestinal cancer who were treatment-naïve and initiated first-line chemotherapy. All 90 patients completed the planned baseline, Month 1, and Month 3 assessments. Baseline evaluation included EORTC QLQ-C30, PSQI, GAD-7, SMMSE, MNA-SF, ECOG PS, and body weight. Longitudinal quality-of-life, patient-reported, and geriatric assessments were repeated at Months 1 and 3.

Supplementary Figure S2. Longitudinal Changes in EORTC QLQ-C30 Global Health Status/Quality of Life During First-Line Chemotherapy

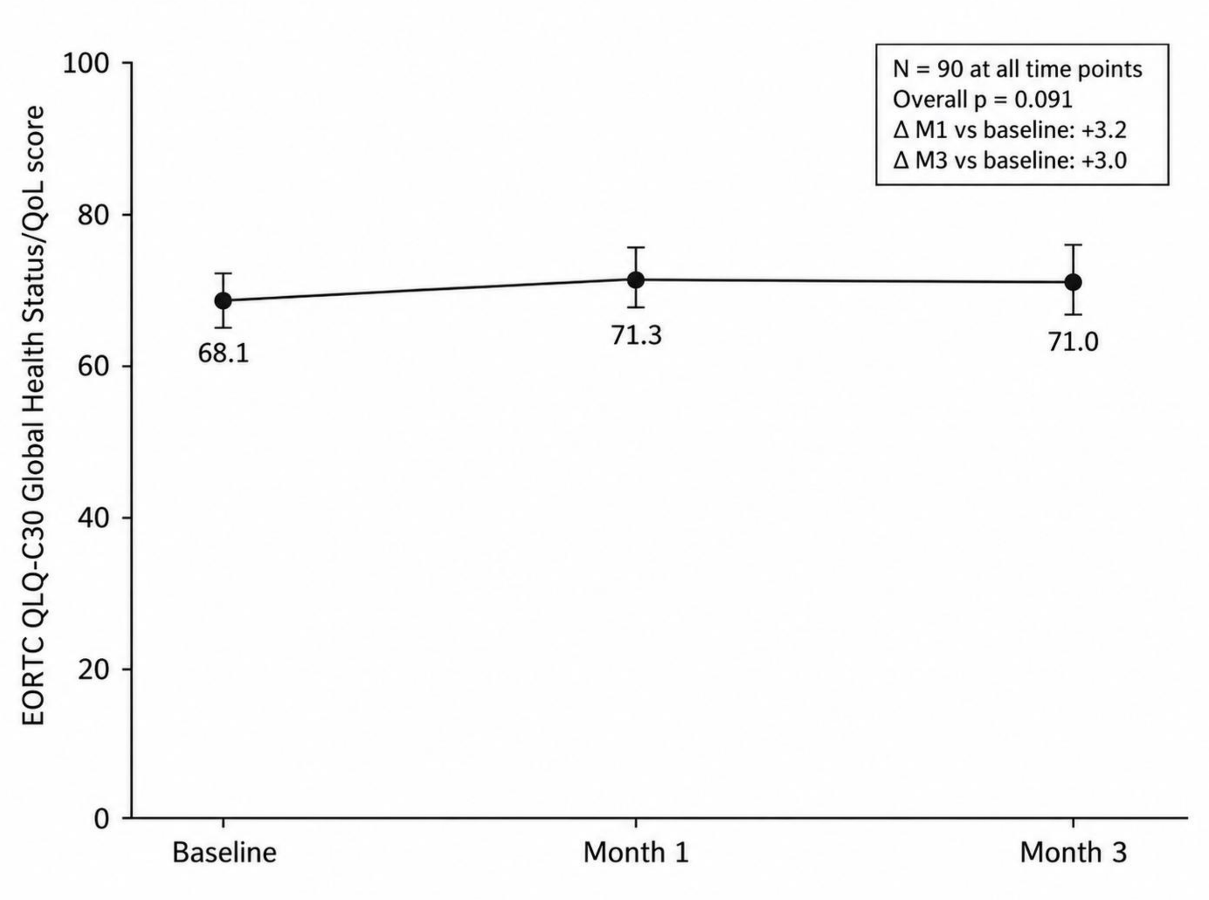

Abbreviations: CI, confidence interval; EORTC QLQ-C30, European Organisation for Research and Treatment of Cancer Quality of Life Questionnaire Core 30; M1, Month 1; M3, Month 3; QoL, quality of life.

Points represent model-estimated mean EORTC QLQ-C30 Global Health Status/QoL scores at baseline, Month 1, and Month 3; error bars indicate 95% confidence intervals. Longitudinal change was evaluated using a linear mixed-effects model with assessment time entered as a categorical fixed effect and patient as a random intercept. All 90 patients had evaluable QoL data at each time point. Mean Global Health Status/QoL scores were 68.1 at baseline, 71.3 at Month 1, and 71.0 at Month 3. The estimated mean changes from baseline were +3.2 points at Month 1 and +3.0 points at Month 3; the overall effect of time was not statistically significant ($p = 0.091$). Higher scores indicate better global health status and quality of life.

Supplementary Figure S3. Longitudinal Changes in Patient-Reported and Geriatric Measures During First-Line Chemotherapy

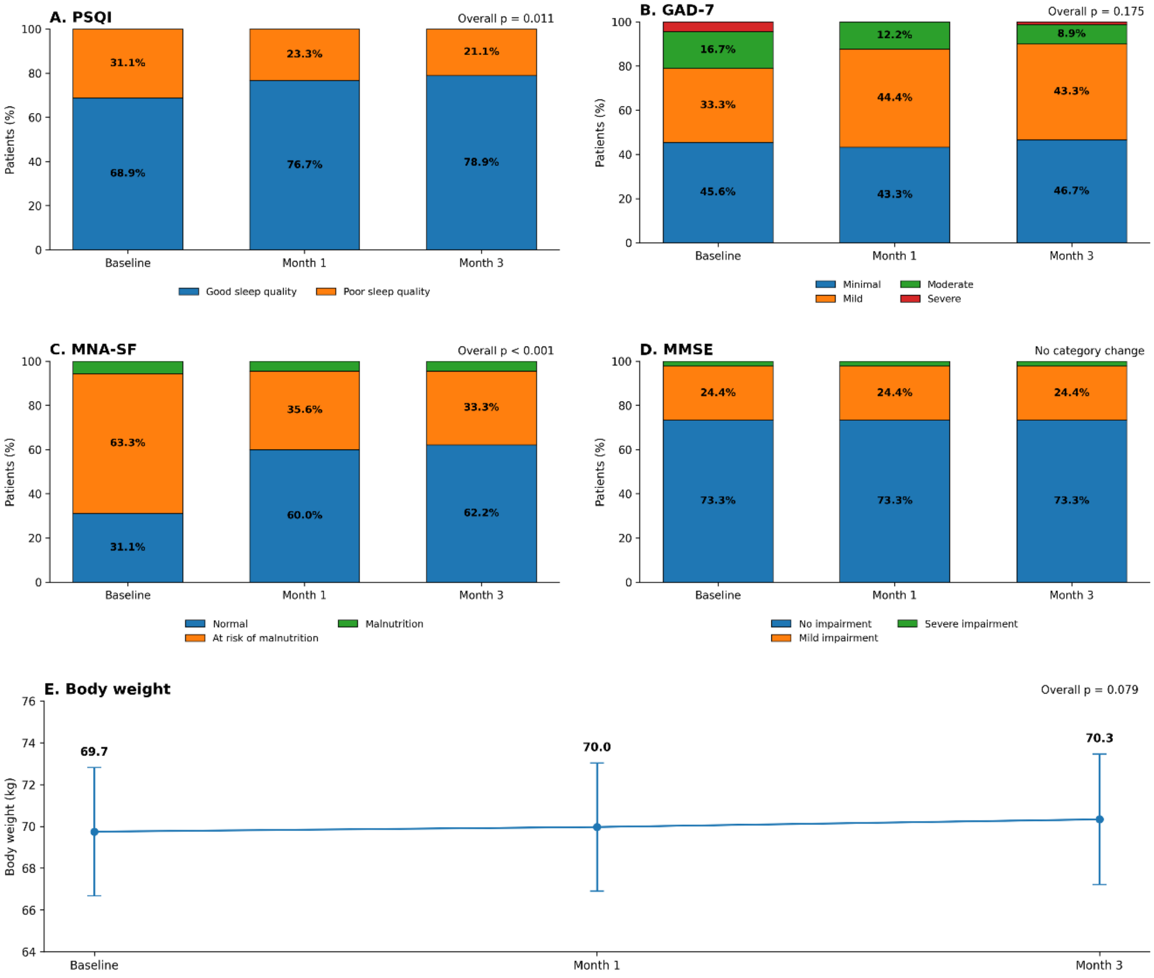

Abbreviations: CI, confidence interval; ECOG PS, Eastern Cooperative Oncology Group performance status; GAD-7, Generalized Anxiety Disorder-7; SMMSE, Standardized Mini-Mental State Examination; MNA-SF, Mini Nutritional Assessment–Short Form; PSQI, Pittsburgh Sleep Quality Index.

Panels A–D show the distribution of categorical patient-reported and geriatric measures at baseline, Month 1, and Month 3. Panel A presents PSQI sleep-quality categories; Panel B, GAD-7 anxiety categories; Panel C, MNA-SF nutritional-status categories; and Panel D, SMMSE cognitive-status categories. Percentages are calculated among all 90 patients at each time point. Overall longitudinal changes were assessed using Cochran's Q test for binary PSQI categories and ordinal longitudinal models for GAD-7 and MNA-SF; SMMSE categories remained unchanged in all patients across the three assessments. Panel E shows mean body weight at baseline, Month 1, and Month 3 with 95% confidence intervals; longitudinal change in body weight was evaluated using a linear mixed-effects model with time as a categorical fixed effect and patient as a random intercept. All 90 patients had available data at each assessment.

Supplementary Figure S4. Kaplan–Meier Curves for Progression-Free and Overall Survival

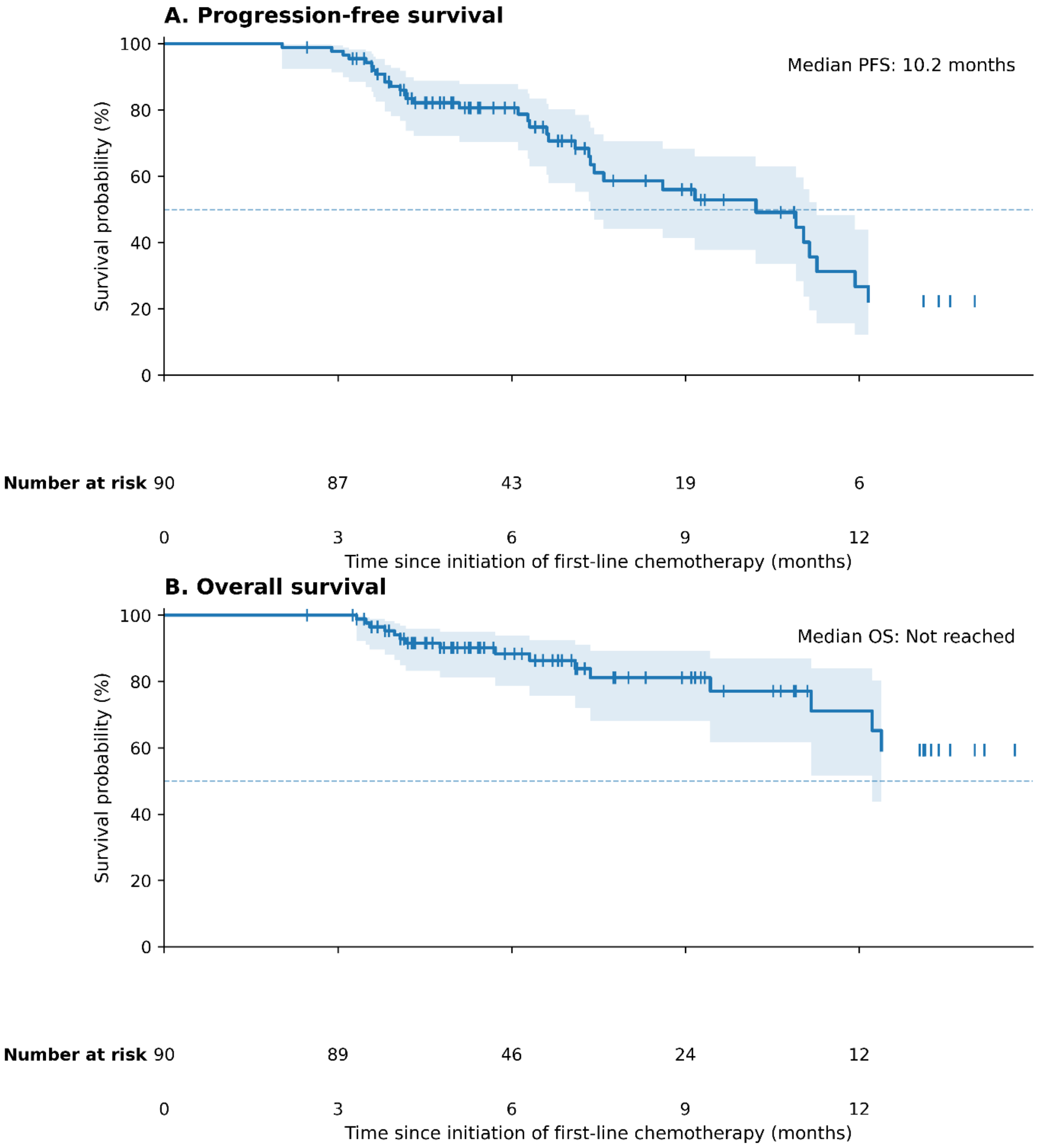

Abbreviations: CI, confidence interval; NR, not reached; OS, overall survival; PFS, progression-free survival.

Kaplan–Meier curves show progression-free survival (Panel A) and overall survival (Panel B) from initiation of first-line chemotherapy. Shaded areas represent 95% confidence intervals, and tick marks indicate censored observations. Numbers at risk are shown at 0, 3, 6, 9, and 12 months. PFS was defined as the interval from initiation of first-line chemotherapy to radiologically documented disease progression or death from any cause, whichever occurred first; patients without a PFS event were censored at the date of last clinical follow-up. OS was defined as the interval from initiation of first-line chemotherapy to death from any cause; patients alive at last follow-up were censored on that date. Among 90 patients, 35 PFS events and 16 deaths occurred. Median PFS was 10.2 months, whereas median OS was not reached at the time of data cutoff.
